# Highly active alkyne metathesis catalysts operating under open air condition

**DOI:** 10.1038/s41467-021-21364-4

**Published:** 2021-02-18

**Authors:** Yanqing Ge, Shaofeng Huang, Yiming Hu, Lei Zhang, Ling He, Sebastian Krajewski, Michael Ortiz, Yinghua Jin, Wei Zhang

**Affiliations:** 1 School of Chemistry and Pharmaceutical Engineering, Shandong First Medical University & Shandong Academy of Medical Sciences, Taian, China; 2 Department of Chemistry, University of Colorado Boulder, Boulder, CO USA; 3 College of Chemistry, Sichuan University, Chengdu, China

**Keywords:** Catalyst synthesis, Homogeneous catalysis, Synthetic chemistry methodology

## Abstract

Alkyne metathesis represents a rapidly emerging synthetic method that has shown great potential in small molecule and polymer synthesis. However, its practical use has been impeded by the limited availability of user-friendly catalysts and their generally high moisture/air sensitivity. Herein, we report an alkyne metathesis catalyst system that can operate under open-air conditions with a broad substrate scope and excellent yields. These catalysts are composed of simple multidentate tris(2-hydroxyphenyl)methane ligands, which can be easily prepared in multi-gram scale. The catalyst substituted with electron withdrawing cyano groups exhibits the highest activity at room temperature with excellent functional group tolerance (-OH, -CHO, -NO_2_, pyridyl). More importantly, the catalyst provides excellent yields (typically >90%) in open air, comparable to those operating under argon. When dispersed in paraffin wax, the active catalyst can be stored on a benchtop under ambient conditions without any decrease in activity for one day (retain 88% after 3 days). This work opens many possibilities for developing highly active user-friendly alkyne metathesis catalysts that can function in open air.

## Abstract (web summary)

Alkyne metathesis catalysts usually suffer from high moisture/air sensitivity, which limit their wide applicability. Here, the authors report efficient alkyne metathesis catalysts that can operate under open-air conditions with a broad functional group tolerance.

## Introduction

**Fig. 1:**
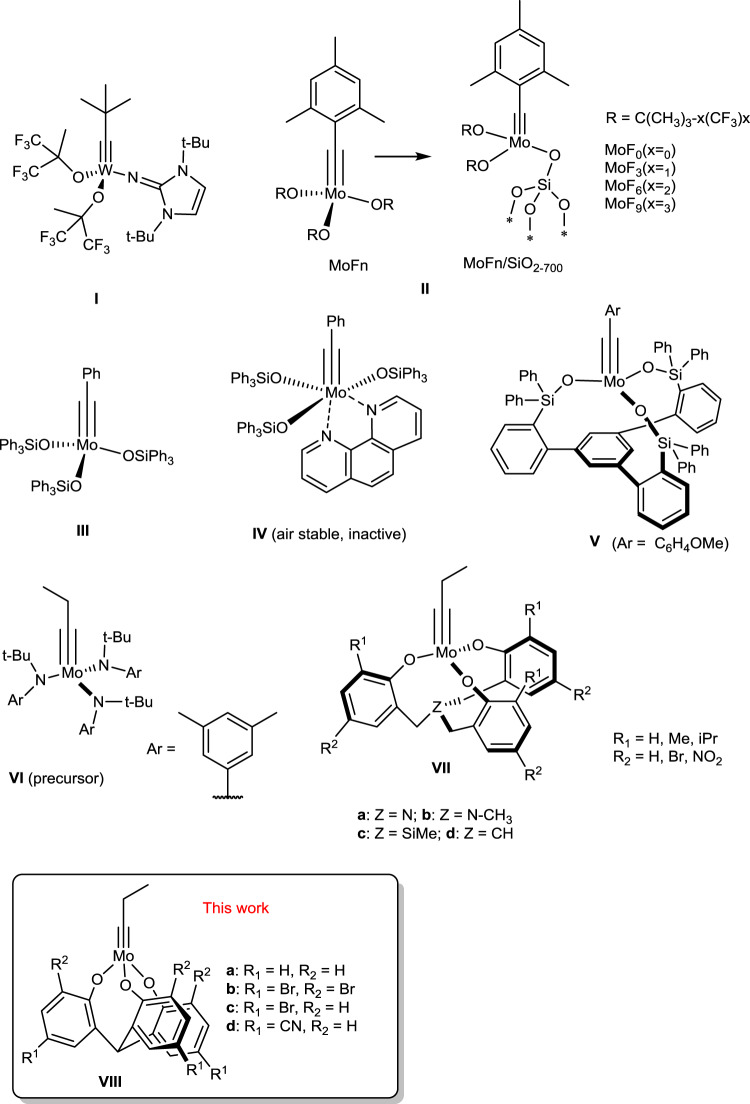
Chemical structures of selected catalysts for alkyne metathesis. Representative examples of previously reported alkyne metathesis catalysts and precursors are shown as **I**-**VII**. The proposed structure of the catalyst system reported in this work is shown as **VIII**.

Alkyne metathesis has been widely applied in the synthesis of natural products^1–5^, polymers^6–19^, shape-persistent macrocycles, and molecular cages^20–28^ since its initial discovery in 1968^29^. Within the past two decades, several efficient and well-defined catalysts containing fluorinated alkoxides, silanolates, or amides ligands have been developed (Fig. 1)^30–35^. For example, Tamm and co-workers introduced the tungsten complex **I** with imidazolin-2-iminato ligands, which is among the first few catalysts operating efficiently at room temperature with a low catalyst loading^30,31^. Recently, they also reported a series of molecular (MoF_n_) and silica-supported (MoF_n_/SiO_2‑700_) alkyne metathesis catalysts **II**, which catalyze the cross metathesis of terminal alkynes under mild conditions^32,33^. Fürstner developed catalyst **III**, which shows excellent activity, remarkable functional group tolerance (even protic groups) and user-friendliness^34,35^. Such catalysts have been widely used in natural product synthesis. The precatalyst (**IV**) is stable in air and becomes metathesis active upon activation^35^. In 2019, Fürstner and co-workers unveiled an unconventional tris(triarylsilanolate)molybdenum alkylidyne catalyst **V**, which shows even better functional group tolerance, albeit with slow reaction rates and inertness towards nitro substituted substrates^36,37^. The most up-to-date report from the Jia Group elucidated a d^2^Re(V)-based catalyst bearing the PO-chelating ligands and a pyridine ligand, which is air and moisture stable. The catalyst was active for various substrates at high temperature under nitrogen^38^.

Our group has a long-standing interest in developing highly active alkyne metathesis catalysts. We have successfully developed multidentate ligand-based molybdenum catalysts (**VII a-d**) that are easily generated in situ by mixing a molybdenum-trisamide alkylidyne precursor (**VI**) with the multidentate ligands^39–42^. Our multidentate catalytic systems exhibit comparable activity and substrate scope to the above mentioned Fürstner’s catalysts. However, similar to all the previous catalysts, they also require inert atmosphere to provide good yields and their reaction rates are relatively slow at room temperature. Alkyne metathesis catalysts that can operate under user-friendly open air conditions with high efficiency and provide high yields for a wide range of substrates are still rare to date^43–46^. In this work, we report a class of multidentate catalysts containing simple tris(2-hydroxyphenyl)methane ligands, which can be used in open air, providing similar yields to those obtained under argon and otherwise identical conditions. The catalyst can be stored on a benchtop under ambient conditions when dispersed in paraffin. The catalyst consisting of the cyano-substituted ligands shows the highest activity, catalyzing the reactions in open air with high efficiency (typically <10-30 min, >90% yield) and excellent functional group tolerance.

## Results and discussion

### Catalyst design, synthesis and characterization

**Fig. 2:**
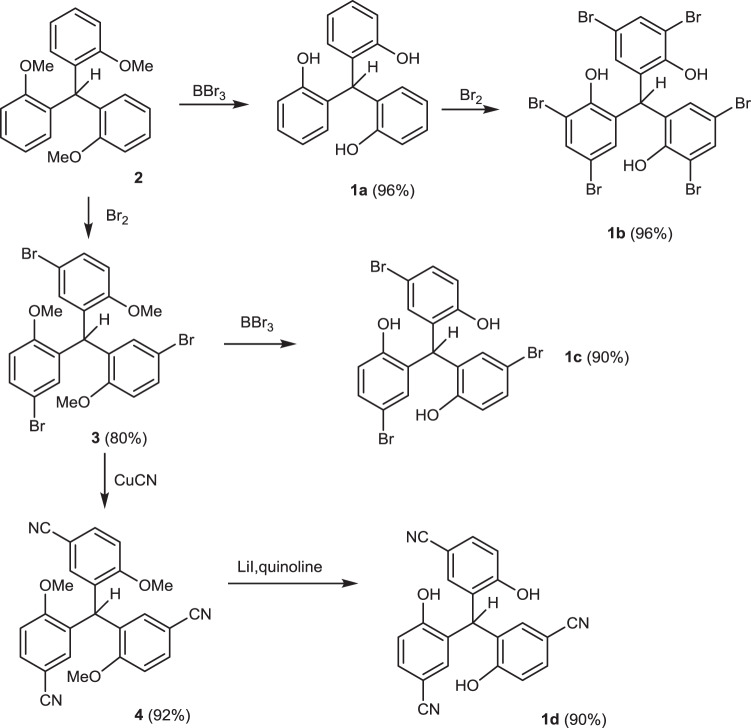
Synthesis of the multidentate ligands **1a–d**. The ligands **1a–d** were synthesized from the same staring material (**2**) in gram-scale within three steps.

**Fig. 3:**
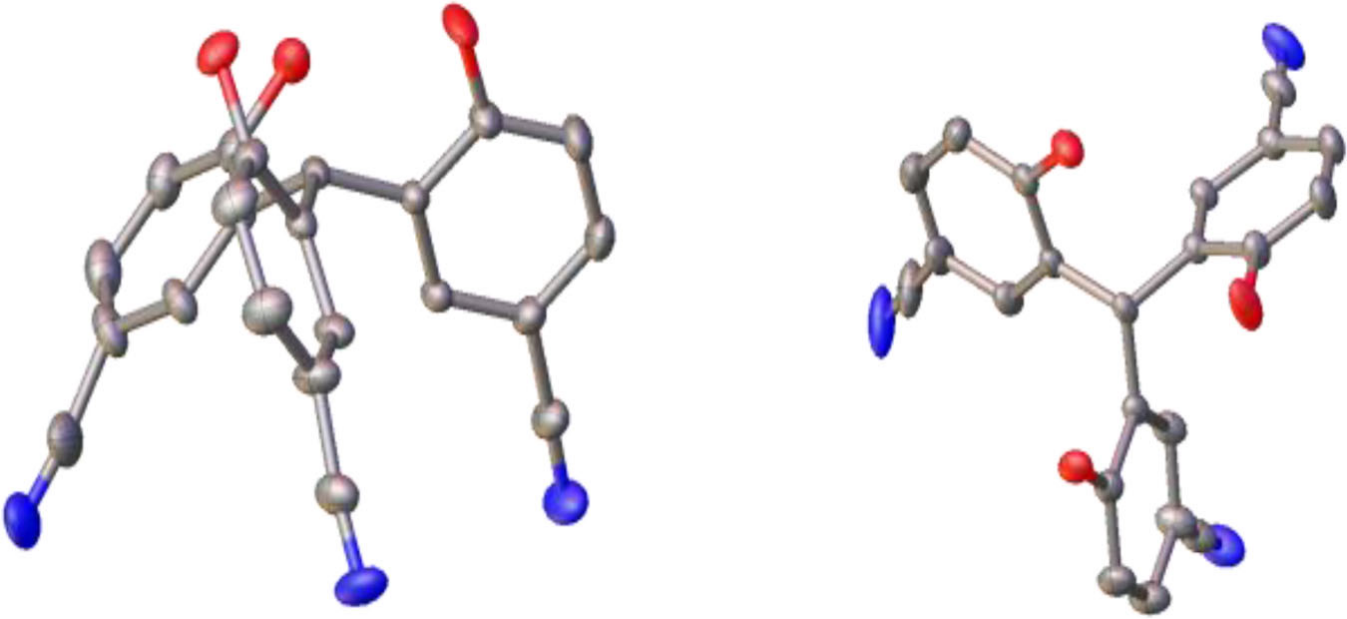
Molecular structure of **1d**. The compound was obtained as single crystal and the structure elucidated through single crystal X-ray analysis (ellipsoids drawn at the 50% probability level, side view (left) and top view (right), Red: O, Gray: C, Blue: N, hydrogen atoms omitted for clarity).

We envisioned that removing the three methylene groups from the ligand **VII** could provide more rigid ligands, which can minimize the configurational entropic cost during the formation of the podand binding motif with the Mo center. Thus, the ligands **1a–d** were prepared from cheap starting materials on multi-gram scales (>5 g) as shown in Fig. 2. The ligands **1a-c** have previously been reported by Yasuda and the literature procedure was followed with some modification^47,48^. The known starting material **2** was prepared in two steps through the addition of aryl lithium to methyl chloroformate followed by *p*-toluenesulfonic acid-mediated deoxygenation of the alcohol. Deprotection of **2** by BBr_3_ effectively afforded the desired ligand **1a**^47^. Bromination of **1a** in AcOH/CCl_4_ gave the ligand **1b**^48^. Ligand **1c** was obtained by bromination of **2** followed by the deprotection of methoxy groups^48^. The ^1^H NMR spectra of **1a-c** were consistent with the literature reports^47,48^. Substitution of **3** with CuCN followed by deprotection yielded the ligand **1d**. The ligand **1d** was characterized by ^1^H NMR, ^13^C NMR, HR-MS, and single crystal X-ray diffraction analysis. Single crystals of compound **1d** (Fig. 3) were obtained by slow evaporation of a solution of **1d** in acetone/ethyl acetate (1:1) at room temperature over two days. The single crystal X-ray analysis of **1d** shows that the three OH groups are “upward”-oriented, which is necessary for the formation of a podand complex. However, it should be noted that the central C-H group is also “upward” oriented, which could cause steric hindrance during the ligand binding with the molybdenum center. The calculation of energy optimized conformers shows that the “upward”-oriented structures, similar to the crystal structure of **1d**, are energy-favored for all four ligands **1a–d**. Although the difference between Gibbs energies of the “upward”-oriented conformers and “downward”-oriented conformers is small (3.7–5.2 kcal/mol), the necessary conformational change could negatively affect the ligand binding to the molybdenum center and the formation of active species.

The active catalysts were generated in situ by mixing precursor **VI** with ligand **1a–d** in 1:1 ratio. After heating a mixture of **1a** and **VI** at 70 °C for 30 min, we observed that the two singlets at 5.8 ppm (broad) and 6.7 ppm corresponding to the aromatic protons of the amido ligands disappeared, accompanying with the increase of the proton signals (two singlets at 6.4 ppm and 6.5 ppm) of the liberated free aniline ligands (Supplementary Information, Supplementary Fig. 1). We also observed the complete shifts of aromatic protons of ligand **1a**. These results indicate the full replacement of the amido ligands with **1a**. We also observed a triplet at -0.8 ppm corresponding to the propylidyne CH_3_ protons, which correlates to a quartet at 1.1 ppm in the COSY spectrum and the carbyne carbon at 317 ppm in the HMBC spectrum (Supplementary Fig. 2 and 3)^41^. We did not observe any other proton signals that have correlation with carbyne carbons. The ^13^C signal of the carbyne carbon of the precursor **VI** shifts downfield from 302 ppm to 317 ppm upon binding of ligand **1a**, further supporting the replacement of the amido ligands with **1a**. It should be noted that more than one molybdenum propylidyne species were observed oftentimes. For example, although ^1^H NMR spectrum of the mixture of **VI** and **1d** (**VIII-d**) shows the presence of only three major proton signals in the aromatic region that clearly belong to the symmetrical ligand **1d**, consistent with the structure of the fully bound **VIII-d**, there are two propylidyne CH_3_ triplets around −0.6 ppm indicating the presence of two molybdenum propylidyne species. However, the existence of more than one molybdenum propylidyne species did not significantly alter the catalytic activities. Our numerous attempts to obtain the crystal structures of the proposed ligand-Mo-propylidyne complexes (**VIII**) and unambiguously determine their structures were not successful. The difficulty in isolating the ligand-Mo-propylidyne complex suggests that the **VIII** structures with all three phenoxide bound to the molybdenum metal center are likely under dynamic equilibrium with other species, e.g., on/off binding of one or two phenolic arms, or formation of molybdenum dimer species.

### Catalytic activity

We examined the activity of these multidentate catalysts consisting of ligands **1a–d**. Alkyne metathesis of 4-propynylbenzaldehyde, a relatively challenging substrate, was used for the model reaction. We conducted the metathesis reactions in a closed system using 5 Å molecular sieves as the 2-butyne scavenger^12^. In a typical procedure, the precursor **VI** and the ligand were mixed in a solvent and heated at 70 °C for 10 min before adding the substrate. The mixture was then reacted at 70 °C for 30 min (3 mol% ligand, 3 mol% precursor, 0.1 mmol 4-propynylbenzaldehyde, 150 mg MS 5 Å, 3 mL CCl_4_). To our great surprise, the catalyst consisting of the unsubstituted ligand **VIII-a** was nearly inactive, showing only 5% yield. It is possible that the ligand **1a** needs to undergo conformational change (flipping the central C-H group) to bind the molybdenum center, causing the low catalytic activity of **VIII-a**. In our previous study, the analogous catalyst **VIId** (R^1^ = R^2^ = H) with extra flexible methylene groups in the core of the ligand showed significantly higher activity (60% yield) for the same substrate (4-propynylbenzaldehyde) under similar conditions (3 mol% catalyst loading, 40 °C, 2 h, CCl_4_). These results appear to contradict with our hypothesis that more rigid ligands can minimize the configurational entropic cost thus would have higher catalytic activity. However, we found the catalyst system **VIII** with more rigid ligand **1a–d** is very sensitive to the electronic effect of the ligand. Although **VIII-a** has very low activity, there was a significant improvement in the catalytic activity of **VIII-b**, **c**, **d** with the enhancement of electron-withdrawing capability of the ligands. The catalyst **VIII-d** consisting of ligand **1d** substituted with electron-withdrawing cyano groups exhibits the highest activity and can catalyze the reaction at room temperature (3 h, 94%) (Table 1, entry 6). The catalyst **VIII-d** even outperforms the best performing catalyst **VIId** (R^1^ = ^*i*^Pr, R^2^ = H). The catalyst **VIII-d** showed higher catalytic activity and faster reaction kinetics compared to **VIId** (R^1^ = ^*i*^Pr, R^2^ = H) in the alkyne metathesis of 1-nitro-4-propynyl benzene (Supplementary Fig. 11a). In addition, **VIII-d** consistently showed higher activity than **VIId** (R^1^ = ^*i*^Pr, R^2^ = H) in the alkyne metathesis of multiple other substrates in air under otherwise identical conditions (Supplementary Fig. 11b). These results suggest the advantages of the catalyst system consisting of the more rigid ligands. It should be noted that **VIII** and **VIId** catalytic systems behave very differently. As we previously reported, the catalyst system **VIId** is very sensitive to the steric bulk of the *ortho* substituents on the ligands but does not show obvious trend with the change of the electronic effect of the ligand substituents. For instance, when more electron-withdrawing nitro groups are attached to the ligand, the catalyst **VIId** (R^1^ = ^*i*^Pr, R^2^ = NO_2_) shows significantly lower activity (two-fold decrease of the conversion) compared to **VIId** (R^1^ = ^*i*^Pr, R^2^ = H) under otherwise identical conditions. On the contrary, the catalyst system **VIII** is very sensitive to the electronic effect of the ligand. For the **VIId** system, the catalyst with the isopropyl substituted ligand (R^1^ = ^*i*^Pr, R^2^ = H) showed the highest activity, whereas for **VIII** system, the catalyst with the cyano substituted ligand (R^1^ = CN, R^2^ = H) showed the highest activity. Due to the dramatic difference in their responses to the steric and electronic effect of the substituents on the ligands, the two catalyst systems (**VIId** and **VIII**) are hard to be directly compared. Therefore, it is unclear how the rigidity of a ligand influences the catalytic activity. Nevertheless, the best catalyst (**VIII-d**) in the **VIII** system consisting of the rigid ligand **1d** has a higher activity than the best catalyst in the more flexible **VIId** system. **VIII-b** and **VIII-c** consisting of the ligands with weak electron withdrawing bromo substituents show much lower catalytic activity (88% and 62%, respectively) under otherwise identical conditions. Given its superior performance, we used **1d** as the catalyst ligand in the following study.

**Table 1:** Catalytic activities of multidentate catalysts consisting of ligands **1a–d**.


Entry^a^	Ligand	Temperature (°C)	Time (h)	Yield (%)^b^
**1**	**1a**	70	0.5	5
**2**	**1b**	70	0.5	88
**3**	**1c**	70	0.5	62
**4**	**1d**	70	0.5	96
**5**	**1d**	40	1.5	94
**6**	**1d**	22	3	94

^a^0.1 mmol substrate, 150 mg MS 5 Å, 3.0 mL CCl_4_, 0.003 mmol precursor, and 0.003 mmol ligand were used.

^b^The yields were estimated based on the proton signal integrations in the crude product ^1^H NMR spectra.

Next, the solvent effect on the catalytic activity of **VIII-d** was examined using 4-propynylbenzaldehyde as the substrate (Supplementary Information, Supplementary Table 1). The activity of the catalyst varies significantly in different solvents. We obtained the highest yield in carbon tetrachloride (96%). The product yields in chloroform (79%) and chlorobenzene (76%) were good, but in other solvents such as toluene and dichloroethane, the yields were much lower (18% and 4%, respectively). Only about 1% of the dimer products were observed in acetone, THF and CH_3_CN. Although the catalyst showed the highest activity in CCl_4_, the toxicity and health safety issues of CCl_4_ would be a concern for large scale synthesis. Therefore, we also explored the possibility of minimizing the use of CCl_4_. We found CCl_4_ is critical for the generation of the active catalyst species but less critical for the metathesis reaction. When the catalyst was generated in a small amount of CCl_4_ first (10 vol % of the total solvent used in the reaction), then subjected to the metathesis reaction in other solvents, the reaction yields were significantly improved (Supplementary Table 2). Notably, there was only a slight difference between the yields of the reaction in pure carbon tetrachloride (96%) and in toluene-carbon tetrachloride (90%) or chlorobenzene-carbon tetrachloride (91%) co-solvent (solvent/CCl_4_, v/v, 9/1). Therefore, toluene or chlorobenzene can be used as the main solvent to avoid the use of a large amount of CCl_4_. The possibility of using other solvents is also beneficial for many substrates with poor solubility in carbon tetrachloride.

**Fig. 4:**
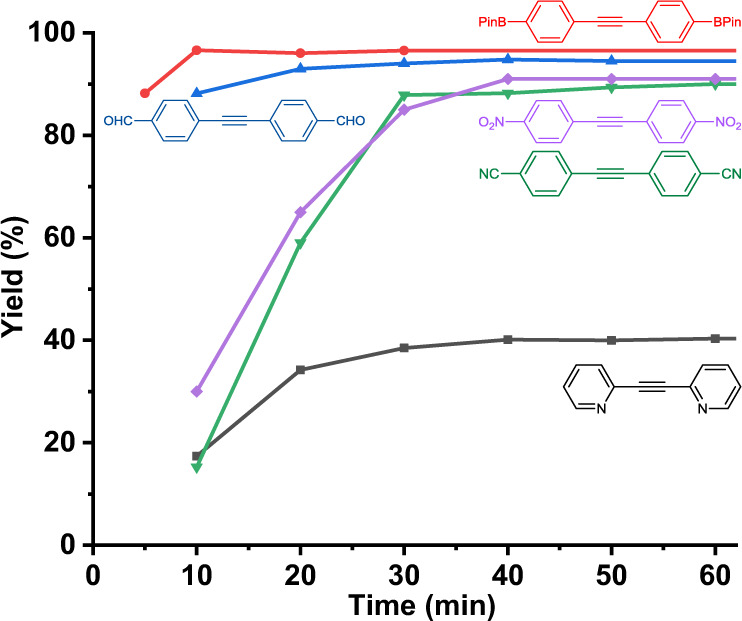
Kinetic studies of the alkyne metathesis using **VIII-d** catalyst. Various substrates were tested. The reaction was monitored using ^1^H NMR spectroscopy and the yield was estimated based on the integration of the proton signals. Conditions: 0.1 mmol substrate, 150 mg 5 Å molecular sieves, 3.0 mL CCl_4_, 0.003 mmol precursor, and 0.003 mmol ligand **1d**.

The kinetic studies showed that catalyst **VIII-d** is highly efficient in CCl_4_ (Fig. 4). The reactions were mostly completed within 30 min at 70 °C even for the electron poor substrates substituted with cyano or nitro groups. The metathesis reaction proceeds significantly faster with the increase of electron density of the substrate alkynyl groups (98% yield within 10 min, Table 2, entry 1, 2, 3). For example, the reaction rate of the even slightly electron-poor boronate substrate is almost 5–6 times faster than the metathesis rate of the cyano-substituted substrate, reaching 90% yield within 5 min. The reaction of 4-methoxy phenylpropyne could be completed within 30 min even at 20  °C.

**Table 2:** Homodimerization, RCAM, and cyclooligomerization reactions of propynyl substrates either under argon or air condition^a^.

^a^Reaction conditions: CCl_4_ was used as the solvent. 3 mol% catalyst was used for entries 1–9 and 2 mol% catalyst was used for entries 10–12. 150 mg MS 5 Å/0.1 mmol substrate was added in all entries except entry 12.

^b^The yields were estimated based on the proton signal integrations in the crude product ^1^H NMR spectra.

^c^The isolated yield obtained after column chromatography purification.

### Substrate scope

We then examined the substrate scope of this catalyst system in carbon tetrachloride. The reactions were catalyzed by the in situ generated catalyst from the precursor and ligand **1d** (3 mol% loading) in the presence of 5 Å molecular sieves at 70 °C for 10-30 min (at rt for 30–90 min). As shown in Table 2, a broad range of substrates went through the metathesis efficiently. The alkyne metathesis of phenylpropyne substrates with a variety of substituents, such as methoxy, borate ester, chloro groups and even challenging examples containing aldehyde, cyano and nitro functional groups, underwent the metathesis reactions in good to excellent yields (entry 1–6, 89–98% yields). Importantly, the catalyst system achieved 89% conversion of the nitro-substituted phenylpropyne substrate, which is the highest reported to date. The ring-closing alkyne metathesis (RCAM) of diyne to cycloalkyne (entry 10, 11), and the precipitation-driven cyclooligomerization of the carbazole diyne substrate (entry 12) were also successfully carried out. The metathesis of substrates containing potentially problematic substituents such as phenol (entry 9, 98%) also brought excellent results. A low yield was observed with the ortho-propynyl substituted pyridine substrate, whose conversion stopped at 40% (entry 8, and Fig. 4). We found the catalyst is not effective in catalyzing terminal alkynes, propargyl-substituted, or trimethylsilyl-substituted alkynes (3% yield) (Supplementary Table 3).

### Alkyne metathesis in open air

**Fig. 5:**
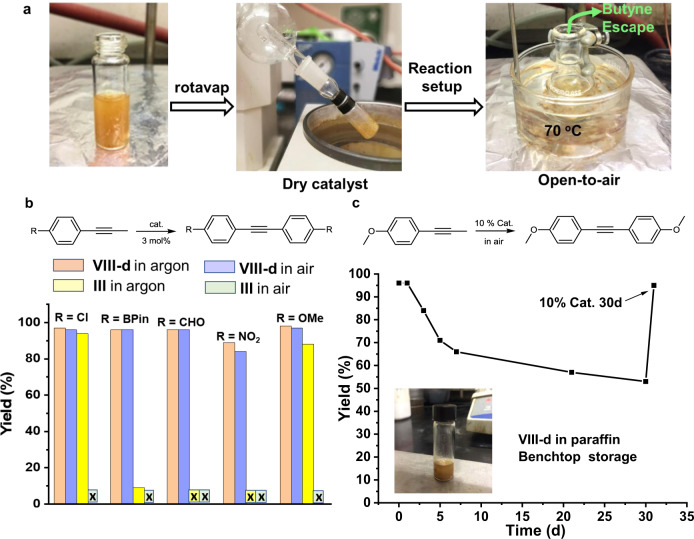
Catalysis in air. **a** Open-air alkyne metathesis using catalyst **VIII-d**. The alkyne metathesis was performed in an open flask at 70 °C in the absence of molecular sieves. The activated catalyst was concentrated to dryness using a rotavap. The whole process took approximately 40 min. **b** Comparison of the catalytic activities of VIII-d and III in air and argon. Conditions for **VIII-d** in open air: 0.1 mmol substrate, 0.003 mmol catalyst (3 mol%), 3 mL CCl_4_, 70 °C, 30 min; Conditions for **VIII-d** in argon: 0.1 mmol substrate, 0.003 mmol catalyst (3 mol%), 3 mL CCl_4_, 150 mg MS 5 Å, 70 °C, 30 min. Conditions for **III** in open air: 0.1 mmol substrate, 0.002 mmol catalyst (2 mol%), 3 mL toluene, 70 °C, 30 min; Conditions for **III** in argon: 0.1 mmol substrate, 0.002 mmol catalyst (2 mol%), 3 mL toluene, 100 mg MS 5 Å, 70 °C, 30 min. **c** Stability of the catalyst dispersed in paraffin wax and stored on a bench top under ambient conditions. 4-Methoxylpropynylbenzene was used as the substrate and the reaction conversion was monitored at different time intervals in air.

As discussed before, the sensitivity of the catalysts to air/moisture represents a key limiting factor, impeding the wide practical applications of alkyne metathesis. Given the excellent performance of this class of multidentate catalysts, subsequently we explored the possibility of running alkyne metathesis under open-air conditions as shown in Fig. 5. We used CCl_4_ in the open-air experiments, which is the best performing solvent and also highly immiscible with water (0.13 g H_2_O/kg CCl_4_, 130 ppm). After mixing the precursor and the ligand **1d** (ratio = 1:1 for activation) in CCl_4_ for 10 min under argon, the catalyst mixture was taken out of the glove box and transferred to a rotavapor. The solvent was removed by rotary evaporation and a solution of the substrate (0.1 mmol) in CCl_4_ (3 mL) was added. The mixture was heated at 70 °C in an open flask for 10–30 min without adding 5 Å molecular sieves. The butyne was removed as a vapor from the open reaction bottle. For all the tested substrates, the yields of the reactions in air were nearly the same as those conducted under argon (Table 2, the last column), indicating the high stability and activity of **VIII-d** catalyst system in open air conditions.

For a few substrates, we also tested Fürstner’s Mo(VI) triphenylsilanolate catalyst **III** (Ar = Ph) in metathesis reactions and compared the activity with **VIII-d**. As shown in Fig. 5b, the alkyne metathesis of chloro-substituted and methoxy-substituted substrates provided similar yields when catalyzed by either **III** or **VIII-d**, under identical conditions (70 °C, 30 min, in the glovebox filled with argon). However, the metathesis of the pinacol boronate (BPin) substituted substrate catalyzed by **III** showed a significantly (more than ten-fold) lower yield (9%) than that (96%) catalyzed by **VIII-d**. The catalyst **III** was inert for the substrates substituted with electron withdrawing nitro and aldehyde groups at room temperature under argon, which is in great contrast to the high yields (96% and 89% respectively) of these reactions under the catalysis of **VIII-d**. In open air, the catalyst **III** was completely inactive for all the five substrates tested. The significantly higher activity of **VIII-d** compared to **III** for multiple substrates with various substituents (electron donating or withdrawing) in both open air and argon can be attributed to the use of multidentate ligand **1d**, which generally has higher cooperative chelating effect compared to the monodentate ligand used in **III**.

To gain a better understanding of the high activity and stability of the catalyst **VIII-d**, we also examined the catalytic activity of the best performing catalyst from our previous flexible trisbenzylmethine system, **VIId** (R^1^ = ^*i*^Pr, R^2^ = H), also consisting of a multidentate ligand. The catalyst **VIId** showed a similar activity (~96% yield) to **VIII-d** under argon for the three tested substrates, methoxy, aldehyde, or pinacol boronate-substituted propynyl benzene. However, significantly decreased yields were observed when the metathesis reactions were performed in open air (Supplementary Fig. 11). The yields of the metathesis reaction under the catalysis of **VIId** (R^1^ = ^*i*^Pr, R^2^ = H) in open air were decreased to 93%, 83%, and 63%, respectively for methoxy, pinacol boronate, and aldehyde substituted propynyl benzenes. The yield of the metathesis of 4-propynylbenzaldehyde in open air under the catalysis of **VIII-d** was 1.5 times (96% vs 63%) higher than that of the reaction conducted using **VIId** (R^1^ = ^*i*^Pr, R^2^ = H) under otherwise identical conditions. Although the catalytic activity of **VIId** in open air is much lower than that of **VIII-d**, it is still significantly higher than the activity of **III** consisting of the monodentate ligands. These results indicate the multidentate rigid ligands are indeed advantageous for improving the stability and activity of the catalyst. Our result represents the rare experimental demonstration of a practical strategy for storing and use of the alkyne metathesis active complex in air, thus making a great stride in developing user-friendly alkyne metathesis catalysts.

To further improve the user friendliness in handling and storage of the catalyst **VIII-d** and make it easy to operate in air, we also explored benchtop storage of the catalyst in paraffin^49^. The activated catalyst powder was dispersed in hot liquid paraffin wax and the mixture was solidified with stirring at room temperature in the glove box. Then the catalyst wax was transferred into air, weighed (~5 mol %), and dissolved in the solvent CCl_4_ (3 mL) before adding 4-methoxypropynylbenzene (0.1 mmol) substrate. The mixture was heated at 70  °C in air for 10 min without the addition of 5 Å molecular sieves. The yield (97%) was similar to that of the reaction strictly conducted under argon (98%). The catalyst wax exhibits high stability in air: its catalytic activity remained the same after 24 h benchtop storage under ambient conditions (Fig. 5c, 96% yield). To our delight, the catalyst provided 53% yield even after being stored in air for one month. When we doubled the catalyst loading (10 mol%), 94% yield was obtained using the one-month aged catalyst.

### Computer modeling

**Fig. 6:**
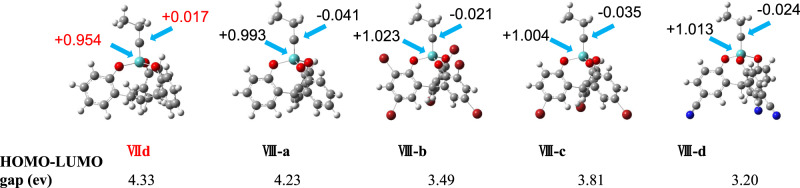
Catalyst structure modeling. Energy minimized structures of the catalytically active species (**VIId**, **VIII-a**, **VIII-b**, **VIII-c**, and **VIII-d**) were optimized at the B3LYP level of density functional theory. The energy gap (HOMO-LUMO gap) between the LUMO energy level of the catalyst and HOMO energy level of the model substrate 1-chloro-4-propynylbenzene was calculated for each catalyst. The Mulliken charge values of the Mo and carbyne carbon (pointed by blue arrows) of each catalyst are shown.

Computer modeling and calculations were also conducted to gain some theoretical understanding of the superior catalytic activity of such triphenylmethane based multidentate catalyst (Fig. 6). Computations were performed by the Gaussian09 suites of programs in this work. Molecular geometries were optimized at the B3LYP level of density functional theory. The SDD basis set was used for Mo, and the 6-31 G** basis set was used for C, N, O and H atoms. The relative values of the atomic charges yielded by population analysis can be used as a parameter to understand the chemical reactivity^50,51^. The Mulliken charge distribution within the Mo-based catalysts was performed at the same level of theory. We also calculated the energy gap between the HOMO of the substrate (1-chloro-4-propynylbenzene) and the LUMO of the catalyst. The calculation showed the advantages of the catalyst with the electron-withdrawing substituents. More electrophilic metal centers with higher Mulliken charge values of +1.023, 1.004, and +1.013 were observed for **VIII-b**, **c**, **d**, compared to the catalyst **VIII-a** (+0.993) (Fig. 6). The higher electrophilicity of molybdenum center would greatly facilitate the initial Mo-alkyne association step. The electrophilicity order (**VIII-b** > **VIII-d** > **VIII-c** > **VIII-a**) of the metal center of the catalyst is roughly consistent with the order of their activity (**VIII-d** > **VIII-b** > **VIII-c** > **VIII-a**). In addition, the electron withdrawing groups of the ligands reduce the LUMO energy of the catalyst, resulting in the lower energy gap between the HOMO of the substrate and the LUMO of the catalyst. The catalyst **VIII-d** shows the lowest HOMO-LUMO energy gap, which is consistent with its highest catalytic activity. The order of the HOMO-LUMO energy gap (**VIII-d** < **VIII-b** < **VIII-c** < **VIII-a**) of the catalysts matches well with the order of their catalytic activity (**VIII-d** > **VIII-b** > **VIII-c** > **VIII-a**). Our previous catalyst **VIId** (R^1^ = R^2^ = H) showed similar HOMO-LUMO energy gap and the electrophilicity of the molybdenum metal center to those of **VIII-a**, which has the lowest activity among the VIII catalyst system. Our calculation results demonstrate the effect of the ligand structure and substituents on the Mulliken charge distribution and the energy gap between the substrate HOMO and the catalyst LUMO on the efficiency of metathesis reactions, which provides useful insights in correlating the catalysts’ structure-activity relationship.

In summary, we developed a class of highly active and robust catalysts, consisting of precursor **VI** and rigid multidentate ligands **1a–d**, for alkyne metathesis reactions. The catalyst **VIII-d** is even active at room temperature and can operate under user-friendly open-air conditions with high efficiency and comparable product yields to those conducted under argon. The catalyst dispersed in paraffin wax shows high stability in air and remains fairly active even after one-month storage in air. The catalysts are tolerant of many organic functional groups, such as phenol, aldehyde, nitro, cyano, and heterocycles, and exhibit high activity. Our study brings chemists a step closer to developing highly active robust user-friendly catalyst system for alkyne metathesis.

## Methods

### Synthesis of tris(2-methoxyphenyl)methanol

The literature procedure was followed with some modification^47^. To a 250 mL Schlenk tube were added anisole (10.7 g, 99.0 mmol), *N*,*N*,*N*’,*N*’- tetramethylethylenediamine (2.31 g, 19.8 mmol), and THF (50 mL). The anisole solution was cooled to 0 °C and a solution of ^*n*^BuLi in hexane (99.0 mmol, 39.6 mL, 2.5 M) was introduced slowly in the Schlenk tube. After stirring for 9 h at room temperature, the Schlenk tube was cooled to 0 °C again and methyl chloroformate (2.85 g, 30.0 mmol) was added slowly. After stirring the reaction mixture overnight at rt, H_2_O (1 L) was added to quench the reaction and the white precipitate was collected by filtration. The crude product (7.68 g, 73%) was used for the next step without further purification.

### Synthesis of compound **2**

The literature procedure was followed with some modification^47^. To a solution of compound tris(2-methoxyphenyl)methanol (3.51 g, 10.0 mmol) in acetonitrile (20 mL) and THF (30 mL) was added TsOH·H_2_O (2.10 g, 11.0 mmol) at 0 °C. After stirring at room temperature for 12 h, H_2_O (200 mL) was added. The solution was extracted with Et_2_O (3 × 50 mL). The combined organic layer was dried (Na_2_SO_4_) and concentrated to give the compound **2** as a yellow solid (3.07 g, 92%).

### Synthesis of ligand **1a**

The literature procedure was followed with some modification^47^. To a solution of compound **2** (3.34 g, 10.0 mmol) in dichloromethane (20 mL) was added BBr_3_ (3.12 mL, 33.0 mmol) at -78 °C. After stirring at rt for 12 h, water (10 mL) was added to the mixture at 0 °C and the white precipitate was collected by filtration. The crude product was recrystallized (hexane/ethyl acetate, v/v = 6/1) to give the pure product **1a** (2.81 g, 96%).

### Synthesis of ligand **1b**

The literature procedure was followed with some modification^48^. To a solution of ligand **1a** (1.17 g, 4.00 mmol) in CCl_4_ (40 mL) was added glacial acetic acid (32 mL) and bromine (5.00 g, 32.0 mmol). The mixture was stirred at rt for 21 h and the white precipitate was collected by filtration. The crude product was recrystallized from acetone/hexane mixture, affording the product as a white solid (2.94 g, 96%).

### Synthesis of tris(5-bromo-2-methoxyphenyl)- methane

The literature procedure was followed with some modification^48^. To a solution of compound **2** (3.35 g, 10.0 mmol) in CCl_4_ (80 mL) was added glacial acetic acid (64 mL) and bromine (12.8 g, 80.0 mmol). The mixture was stirred at room temperature for 20 h and the white precipitate was collected by filtration. The precipitate was dissolved in CH_2_Cl_2_ (200 mL), washed with H_2_O (50 mL), dried over Na_2_SO_4_ and concentrated in vacuo. The crude product (4.58 g, 80%) was used for the next step without further purification.

### Synthesis of ligand **1c**

The literature procedure was followed with some modification^48^. To the solution of compound **3** (5.71 g, 10.0 mmol) in dichloromethane (50 mL) was added BBr_3_ (3.20 mL, 33.0 mmol) at −78 °C. After stirring at room temperature for 48 h, the mixture was poured into 100 mL of ice/water. The mixture was stirred for 1 h and the white precipitate was collected by filtration. The crude product was purified by column chromatography (hexane/ethyl acetate = 50:50) to give the product (4.77 g, 90%) as a white solid.

### Synthesis of 3,3’,3”-methanetriyltris(4- methoxybenzonitrile)

To the solution of compound **3** (5.71 g, 10.0 mmol) in DMF (70 mL) was added CuCN (4.50 g, 50.0 mmol). After stirring at 156 °C for 12 h, FeCl_3_ solution (20 mL in 1 mol/L HCl) was added to the mixture at 60 °C and stirred for 1 h. The mixture was poured into water (600 mL) and the white precipitate was collected by filtration. The crude product (3.77 g, 92%) was used for the next step without further purification.

### Synthesis of ligand **1d**

Compound **4** (2.05 g, 5.0 mmol) and LiI (2.50 g, 150 mmol) were added to quinoline (30 mL) and the mixture was heated at 170 °C for 1 h. 2 N HCl (300 mL) was added to precipitate out the product. The product was redissolved in 0.1 M NaOH and washed with dichloromethane. Neutralization of the aqueous layer with 2 N HCl precipitated out the product as a white solid (1.65 g, 90%).

## Supplementary information

Supplementary Information

## Data Availability

Experimental procedure and characterization data of new compounds are available in Supplementary Information. The X-ray crystallographic data for ligand 1d reported in this study has been deposited at the Cambridge Crystallographic Data Centre (CCDC) under deposition numbers CCDC 1974825. These data can be obtained free of charge from The Cambridge Crystallographic Data Centre via www.ccdc.cam.ac.uk/data_request/cif. Any further relevant data are available from the authors upon reasonable request.
