# Supplementary material for: Highly active alkyne metathesis catalysts operating under open air condition
Source: Nat Commun. 2021 Feb 18;12:1136. doi: 10.1038/s41467-021-21364-4 (PMC7893043; doi:10.1038/s41467-021-21364-4)
Supplement: Supplementary file 1 — Supplementary Information [file 41467_2021_21364_MOESM1_ESM.pdf]

Supplementary Information

Highly Active Alkyne Metathesis Catalysts Operating Under Open Air Condition

Table of Contents

1. Materials and general experimental techniques S2
2. Synthetic procedures S2
   2.1. Synthesis of ligand 1a S2
   2.2 Synthesis of ligand 1b S3
   2.3 Synthesis of ligand 1c S3
   2.4 Synthesis of ligand 1d S4
   2.5 Procedure for metathesis experiments S5
3. *In situ* generation of the catalyst complexes S6
4. Studies on the solvent effect S11
5. Kinetic study of the alkyne metathesis catalyzed by the catalyst VIII-d S12
6. Catalytic activity comparison between catalysts VIII-d and VIId (R^1 = ^{i}Pr, R^2 = H). S15
7. Alkyne metathesis with different alkynes S15
8. Computational calculations S16
9. ^{1}H and ^{13}C NMR spectra for selected compounds S17
10. X-ray Crystal Structure Analysis of Compound 1d (CCDC1974825) S37
11. Supplementary References S38

1. Materials and general experimental techniques

Reagents and solvents were purchased from commercial suppliers and used without further purification, unless otherwise indicated. Ether, tetrahydrofuran, toluene, CH_2Cl_2 and DMF are purified by MBRAUN solvent purification systems. Reagent-grade $CHCl_3$ was purchased from Sigma-Aldrich and was stabilized by amylene, CCl_4 was purchased from Acros, and other solvents were purchased from Fischer Scientific. All the solvents for metathesis reactions were dried with activated 4Å molecular sieves overnight before use. The molybdenum trisamide precursor (**VI**), **VIId (R^1 = ^{i}Pr, R^2 = H)**, and the molybdenum triphenylsilanolate catalyst (Ar = Ph, **III**) were prepared following the reported procedure.[1-3] All metathesis reactions were run under an atmosphere of argon, unless otherwise specified.

The ^{1}H and ^{13}C NMR spectra were recorded on Bruker AV-III 300 MHz spectrometer at 293 K using $CDCl_3$, Acetone-d_6 or DMSO-d_6 as solvent, and tetramethylsilane (TMS) as an internal standard. The HMBC Spectrum was acquired on a Varian INOVA-500 NMR spectrometer using the VNMRJ 3.2A instrument software (Agilent Technologies).

The high-resolution mass spectra were obtained on Waters SYNAPT G2 High Definition Mass Spectrometry System.

2. Synthetic procedures

2.1. Synthesis of ligand 1a

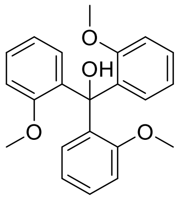

Synthesis of tris(2-methoxyphenyl)methanol: The literature procedure was followed with some modification.[4] To a 250 mL Schlenk tube were added anisole (10.7 g, 99.0 mmol), *N*,*N*,*N'*,*N'*- tetramethylethylenediamine (2.31 g, 19.8 mmol), and THF (50 mL). The anisole solution was cooled to 0 ºC and a solution of ^{n}BuLi in hexane (99.0 mmol, 39.6 mL, 2.5 M) was introduced slowly in the Schlenk tube. After stirring for 9 h at rt, the Schlenk tube was cooled to 0 ºC again and methyl chloroformate (2.85 g, 30.0 mmol) was added slowly. After stirring the reaction mixture overnight at rt, H_2O (1 L) was added to quench the reaction and the white precipitate was collected by filtration. The crude product (7.68 g, 73%) was used for the next step without further purification. The physical data of the product: ^{1}H NMR: (300 MHz, $CDCl_3$) 7.20-7.10 (m, 6H), 6.84-6.79 (m, 6H), 3.39 (s, 9H); ^{13}C NMR: (75 MHz, $CDCl_3$) 157.5, 133.7, 129.8, 128.2, 120.2, 112.4, 80.3, 55.6. The NMR data is consistent with the literature report by M.Yasuda.[4]

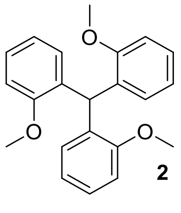

2

Synthesis of compound 2: The literature procedure was followed with some modification.[4] To a solution of compound tris(2-methoxyphenyl)methanol (3.51 g, 10.0 mmol) in acetonitrile (20 mL) and THF (30 mL) was added TsOH·H_2O (2.10 g, 11.0 mmol) at 0 ºC. After stirring at rt

for 12 h, H_2O (200 mL) was added. The solution was extracted with Et_2O (3 x 50 mL). The combined organic layer was dried (Na_2SO_4) and concentrated to give the compound **2** as a yellow solid (3.07 g, 92%). The physical data of the product **2**: ^1H NMR: (300 MHz, $CDCl_3$) 7.24-7.19 (m, 3H), 6.90-6.76 (m, 9H), 6.45 (s, 1H), 3.71 (s, 9H); ^{13}C NMR: (75 MHz, $CDCl_3$) 157.4, 132.6, 129.7, 127.0, 120.0, 110.8, 55.8, 37.0. The NMR data is consistent with the literature report by M.Yasuda.[4]

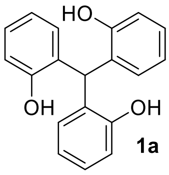

1a

Synthesis of ligand 1a: The literature procedure was followed with some modification.[4] To a solution of compound **2** (3.34 g, 10.0 mmol) in dichloromethane (20 mL) was added BBr_3 (3.12 mL, 33.0 mmol) at -78 ºC. After stirring at rt for 12 h, water (10 mL) was added to the mixture at 0 ºC and the white precipitate was collected by filtration. The crude product was recrystallized (hexane/ethyl acetate, v/v = 6/1) to give the pure product **1a** (2.81 g, 96%). The physical data of the product **1a**: ^1H NMR: (300 MHz, $CDCl_3$) 7.25-7.19 (m, 3H), 6.94-6.85 (m, 9H), 6.09 (s, 1H), 4.87 (s, 3H); ^{13}C NMR: (75 MHz, DMSO-d_6) 155.3, 130.9, 129.8, 127.0, 118.6, 115.2, 37.0. HRMS (ESI): calcd. For $C_{19}H_{16}O_3Na$ [M+Na$^+$]: 315.0997. Found 315.1010. The NMR data is consistent with the literature report by M.Yasuda.[4]

2.2 Synthesis of ligand 1b

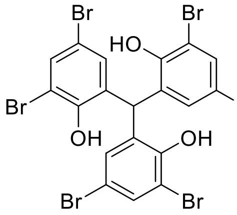

1b

The literature procedure was followed with some modification.[5] To a solution of ligand **1a** (1.17 g, 4.00 mmol) in CCl_4 (40 mL) was added glacial acetic acid (32 mL) and bromine (5.00 g, 32.0 mmol). The mixture was stirred at rt for 21 h and the white precipitate was collected by filtration. The crude product was recrystallized from acetone/hexane mixture, affording the product as a white solid (2.94 g, 96%). The physical data of the product **1b**: ^1H NMR: (300 MHz, DMSO-d_6) 9.55 (s, 3H), 7.67 (d, J = 2.1 Hz, 3H), 6.65 (d, J = 2.1 Hz, 3H), 6.31 (s, 1H); ^{13}C NMR: (75 MHz, DMSO-d_6) 151.6, 134.1, 133.5, 131.1, 112.9, 111.5. HRMS (ESI): calcd. For $C_{19}H_{10}Br_6O_3Na$ [M+Na$^+$]: 788.5568. Found 788.5573. The NMR data is consistent with the literature report by M.Yasuda.[4]

2.3 Synthesis of ligand 1c

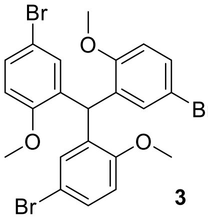

3

Synthesis of tris(5-bromo-2-methoxyphenyl)methane: The literature procedure was followed with some modification.[5] To a solution of compound **2** (3.35 g, 10.0 mmol) in CCl_4 (80 mL) was added glacial acetic acid (64 mL) and bromine (12.8 g, 80.0 mmol). The mixture was stirred at rt for 20 h and the white precipitate was collected by filtration. The precipitate was dissolved in CH_2Cl_2 (200 mL), washed with H_2O (50 mL), dried over Na_2SO_4 and concentrated in vacuo. The crude product (4.58 g, 80%) was used for the next step without further purification. The physical data of the product **3**: ^{1}H NMR: (300 MHz, DMSO-d_6) 7.44 (dd, J = 6.0, 3.0 Hz, 3H), 7.01 (d, J = 9.0 Hz, 3H), 6.65 (d, J = 3.0 Hz, 3H), 6.18 (s, 1H), 3.67 (s, 9H); ^{13}C NMR: (75 MHz, $CDCl_3$) 156.3, 133.3, 131.9, 130.5, 112.8, 112.6, 55.9, 37.4. The NMR data is consistent with the literature report by M.Yasuda.[4]

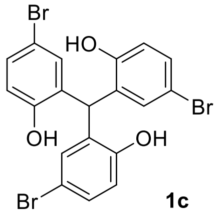

1c

Synthesis of ligand 1c: The literature procedure was followed with some modification.[5] To the solution of compound **3** (5.71 g, 10.0 mmol) in dichloromethane (50 mL) was added BBr_3 (3.20 mL, 33.0 mmol) at -78 ºC. After stirring at rt for 48 h, the mixture was poured into 100 mL of ice/water. The mixture was stirred for 1 h and the white precipitate was collected by filtration. The crude product was purified by column chromatography (hexane/ethyl acetate = 50:50) to give the product (4.77 g, 90%) as a white solid. The physical data of the product **1c**: ^{1}H NMR: (300 MHz, DMSO-d_6) 9.71 (s, 3H), 7.24 (dd, J = 8.7, 2.7 Hz, 3H), 6.78 (d, J = 8.7 Hz, 3H), 6.58 (d, J = 2.7 Hz, 3H), 6.03 (s, 1H); ^{13}C NMR: (75 MHz, DMSO-d_6) 154.7, 132.2, 131.5, 130.5, 117.7, 110.2, 37.9. HRMS (ESI): calcd. For $C_{19}H_{13}Br_3O_3Na$ [M+Na$^+$]: 548.8313. Found 548.8309. The NMR data is consistent with the literature report by M.Yasuda.[4]

2.4 Synthesis of ligand 1d

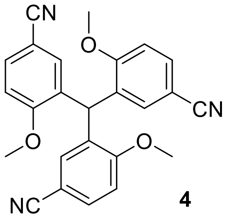

4

Synthesis of 3,3',3''-methanetriyltris(4-methoxybenzonitrile): To the solution of compound **3** (5.71 g, 10.0 mmol) in DMF (70 mL) was added CuCN (4.50 g, 50.0 mmol). After stirring at 156 ºC for 12 h, $FeCl_3$ solution (20 mL in 1 mol/L HCl) was added to the mixture at 60 ºC and stirred for 1 h. The mixture was poured into water (600 mL) and the white precipitate was collected by filtration. The crude product (3.77 g, 92%) was used for the next step without further purification. The physical data of the product **4**: ^{1}H NMR: (300 MHz, DMSO-d_6) 7.79 (dd, J = 8.7, 2.1 Hz, 3H), 7.20 (d, J = 8.7 Hz, 3H), 7.06 (d, J = 2.1 Hz, 3H), 6.27 (s, 1H); ^{13}C NMR: (75 MHz, DMSO-d_6) 160.6, 133.9, 133.0, 131.1, 119.7, 112.6, 103.1, 56.8, 36.2.

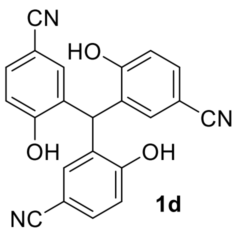

1d

Synthesis of ligand 1d: Compound **4** (2.05 g, 5.0 mmol) and LiI (2.50 g, 150 mmol) were added to quinoline (30 mL) and the mixture was heated at 170 ºC for 1 h. 2 N HCl (300 mL) was added to precipitate out the product. The product was redissolved in 0.1 M NaOH and washed with dichloromethane. Neutralization of the aqueous layer with 2 N HCl precipitated out the product as a white solid (1.65 g, 90%). The physical data of the product **1d**: ^{1}H NMR: (300 MHz, DMSO-d_6) 10.70 (s, 3H), 7.58 (dd, J = 6.0, 3.0 Hz, 3H), 6.97 (m, 6H), 6.13 (s, 1H,); ^{13}C NMR: (75 MHz, DMSO-d_6) 159.7, 133.5, 133.1, 130.1, 120.1, 116.4, 101.3, 36.9. HRMS (ESI): calcd. For $C_{22}H_{13}N_3O_3Na$ [M+Na$^+$]: 390.0855. Found 390.0856.

2.5 Procedure for metathesis experiments

2.5.1 Homodimerization in the glovebox

The ligand **1d** (1.1 mg, 0.003 mmol) and the precursor (2.0 mg, 0.003 mmol) were mixed in dry carbon tetrachloride (2 mL) and stirred for 10 minutes at 70 ºC to generate the catalyst *in situ.* To the generated catalyst solution was added the solution of the substrate (0.1 mmol) in CCl_4 (1 mL) and 5 Å molecular sieves (150 mg). The stirring was continued for 10 - 30 min. The yields were estimated based on the proton signal integrations in the crude product ^{1}H NMR spectra.

2.5.2 Ring closing alkyne metathesis in the glovebox

The ligand **1d** (0.7 mg, 0.002 mmol) and the precursor (1.4 mg, 0.002 mmol) were mixed in dry carbon tetrachloride (2 mL) and stirred for 10 minutes at 70 ºC to generate the catalyst *in situ.* To the generated catalyst solution was added the solution of the substrate (0.1 mmol) in CCl_4 (1 mL) and 5 Å molecular sieves (150 mg). The stirring was continued for 10 min. The yields were estimated based on the proton signal integrations in the crude product ^{1}H NMR spectra.

2.5.3 The precipitation-driven metathesis in the glovebox

The ligand **1d** (0.7 mg, 0.002 mmol) and the precursor (1.4 mg, 0.002 mmol) were mixed in dry carbon tetrachloride (2 mL) and stirred for 10 minutes at 70 ºC to generate the catalyst *in situ.* To the generated catalyst solution was added the solution of the substrate (0.1 mmol) in CCl_4 (1 mL). The stirring was continued for 10 min. The yields were estimated based on the proton signal integrations in the crude product ^{1}H NMR spectra.

2.5.4 Homodimerization in the air

The ligand **1d** (1.1 mg, 0.003 mmol) and the precursor (2.0 mg, 0.003 mmol) were mixed in dry carbon tetrachloride (2 mL) and stirred for 10 minutes under argon at 70 ºC to generate the catalyst *in situ.* The catalyst mixture was taken out of the glove box and transferred to a

rotavapor. The solvent was removed by rotary evaporation and a solution of the substrate (0.1 mmol) in CCl_4 (3 mL) was added. The mixture was heated at 70 ºC in an open flask for 10-30 min without adding 5 Å molecular sieves. The yields were estimated based on the proton signal integrations in the crude product ^{1}H NMR spectra.

2.5.5 Ring closing alkyne metathesis in the air

The ligand **1d** (0.7 mg, 0.002 mmol) and the precursor (1.4 mg, 0.002 mmol) were mixed in dry carbon tetrachloride (2 mL) and stirred for 10 minutes under argon at 70 ºC to generate the catalyst *in situ.* The catalyst mixture was taken out of the glove box and transferred to a rotavapor. The solvent was removed by rotary evaporation and a solution of the substrate (0.1 mmol) in CCl_4 (3 mL) was added. The mixture was heated at 70 ºC in an open flask for 10 min without adding 5 Å molecular sieves. The yields were estimated based on the proton signal integrations in the crude product ^{1}H NMR spectra.

2.5.6 The precipitation-driven metathesis in the air

The ligand **1d** (0.7 mg, 0.002 mmol) and the precursor (1.4 mg, 0.002 mmol) were mixed in dry carbon tetrachloride (2 mL) and stirred for 10 minutes under argon at 70 ºC to generate the catalyst *in situ.* The catalyst mixture was taken out of the glove box and transferred to a rotavapor. The solvent was removed by rotary evaporation and a solution of the substrate (0.1 mmol) in CCl_4 (3 mL) was added. The mixture was heated at 70 ºC in an open flask for 10 min without adding 5 Å molecular sieves. The yields were estimated based on the proton signal integrations in the crude product ^{1}H NMR spectra.

2.5.7 Benchtop storage of the catalyst in paraffin wax

The ligand **1d** (1.1 mg, 0.003 mmol) and the precursor (2.0 mg, 0.003 mmol) were mixed in dry carbon tetrachloride (2 mL) and stirred for 10 minutes under argon at 70 ºC to generate the catalyst *in situ* and then solvent was removed. Hot liquid paraffin wax was added and the mixture was solidified with stirring at room temperature in the glove box. Then the catalyst wax was transferred into air and stored in the air for 30 days.

3. ***In situ*** generation of the catalyst complexes

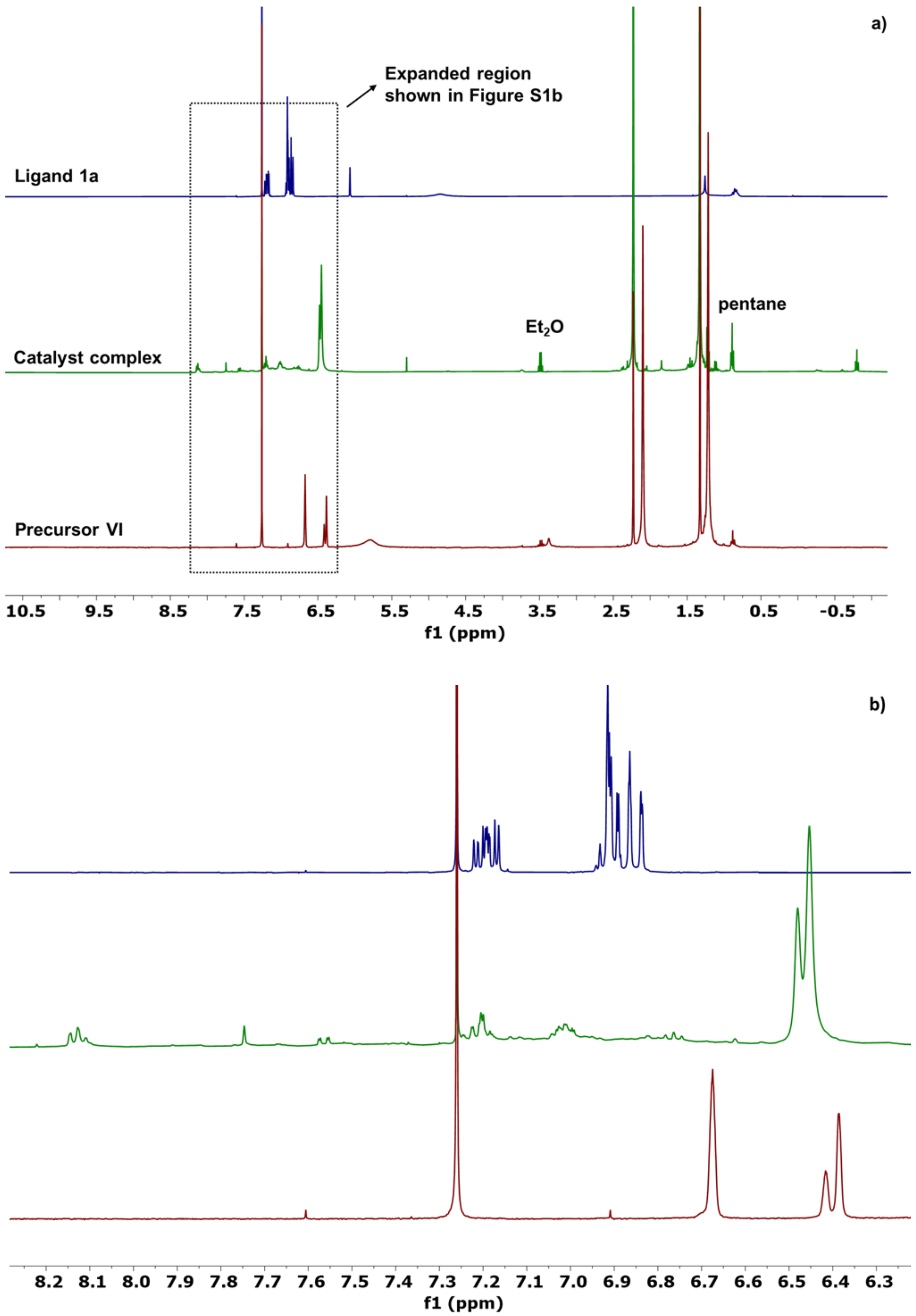

Supplementary Fig. 1. (a) ^{1}H NMR spectra of ligand **1a**, molybdenum precursor **VI**, and the catalyst obtained from the ligand **1a** and **VI** (20 ºC); (b) The NMR of the expanded region shown in the Fig. S1a. $CDCl_3$ was used as the NMR solvent. The catalyst was obtained by heating a mixture of **1a** and **VI** in 1:1 molar ratio in $CDCl_3$ at 70 °C for 30 min.

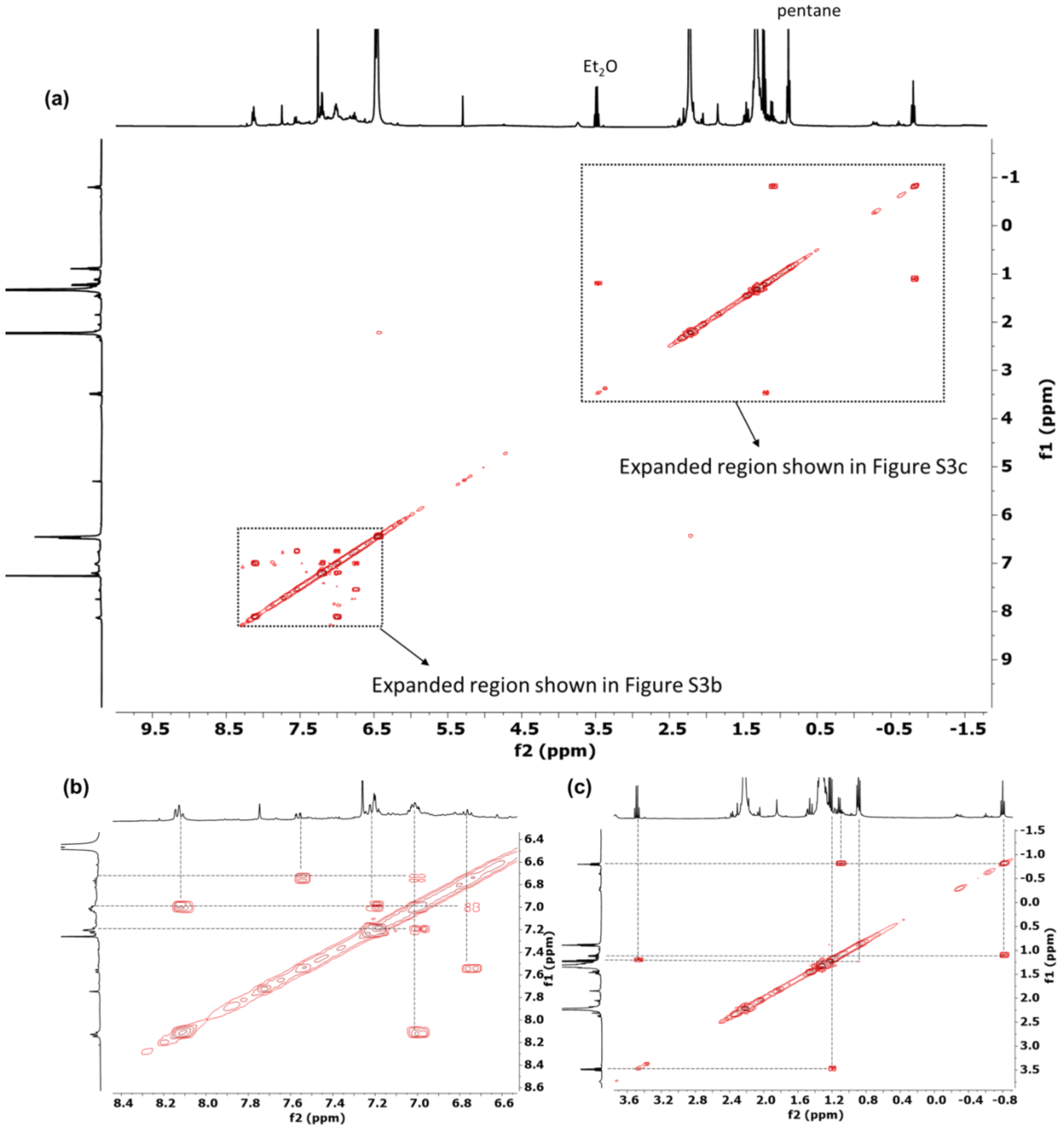

Supplementary Fig. 2. COSY spectra of the catalyst obtained after mixing ligand **1a** and **VI** (20 ºC). $CDCl_3$ was used as the NMR solvent.

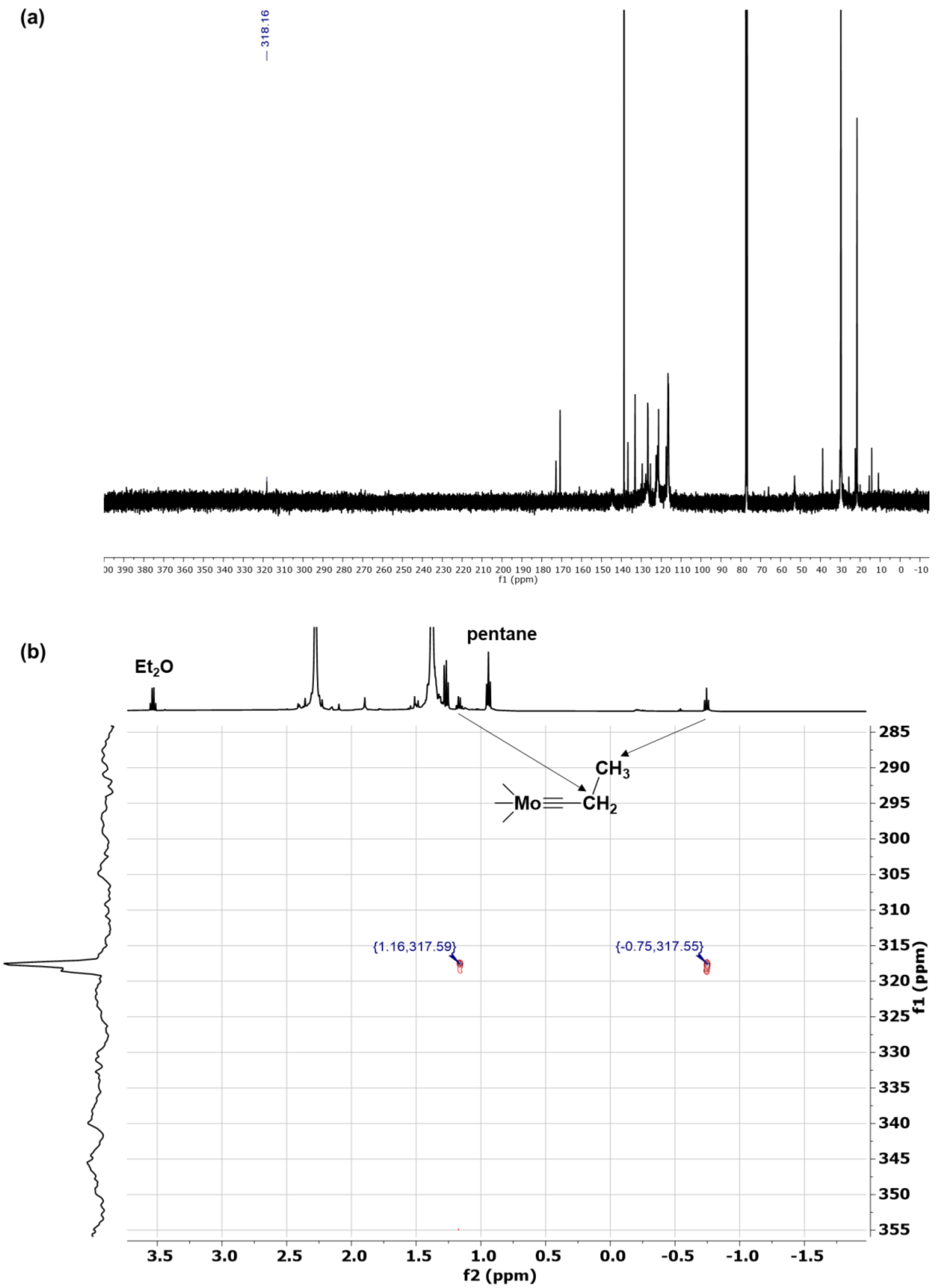

Supplementary Fig. 3. ^{13}C NMR spectrum (a) and HMBC spectrum (b) of the catalyst obtained after mixing ligand **1a** and **VI** (20 ºC) in $CDCl_3$. High resolution band-selective gradient HMBC experiment was conducted, selecting only ^{1}H-^{13}C correlations for carbons appearing between 180-400 ppm.

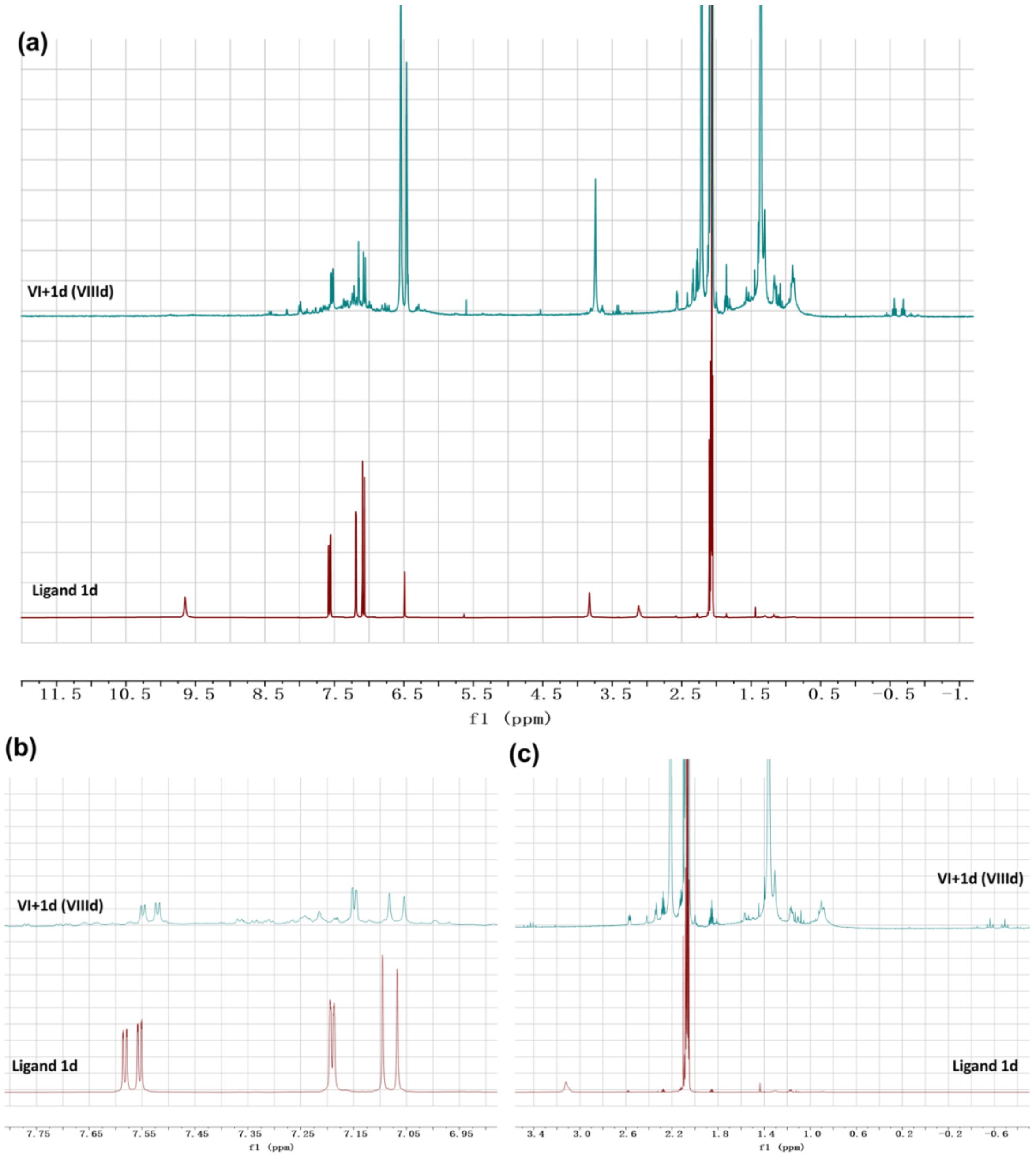

Supplementary Fig. 4. ^{1}H NMR spectra of ligand **1d** and **VIII-d** in deuterated acetone. (b) and (c) show the expanded area of (a).

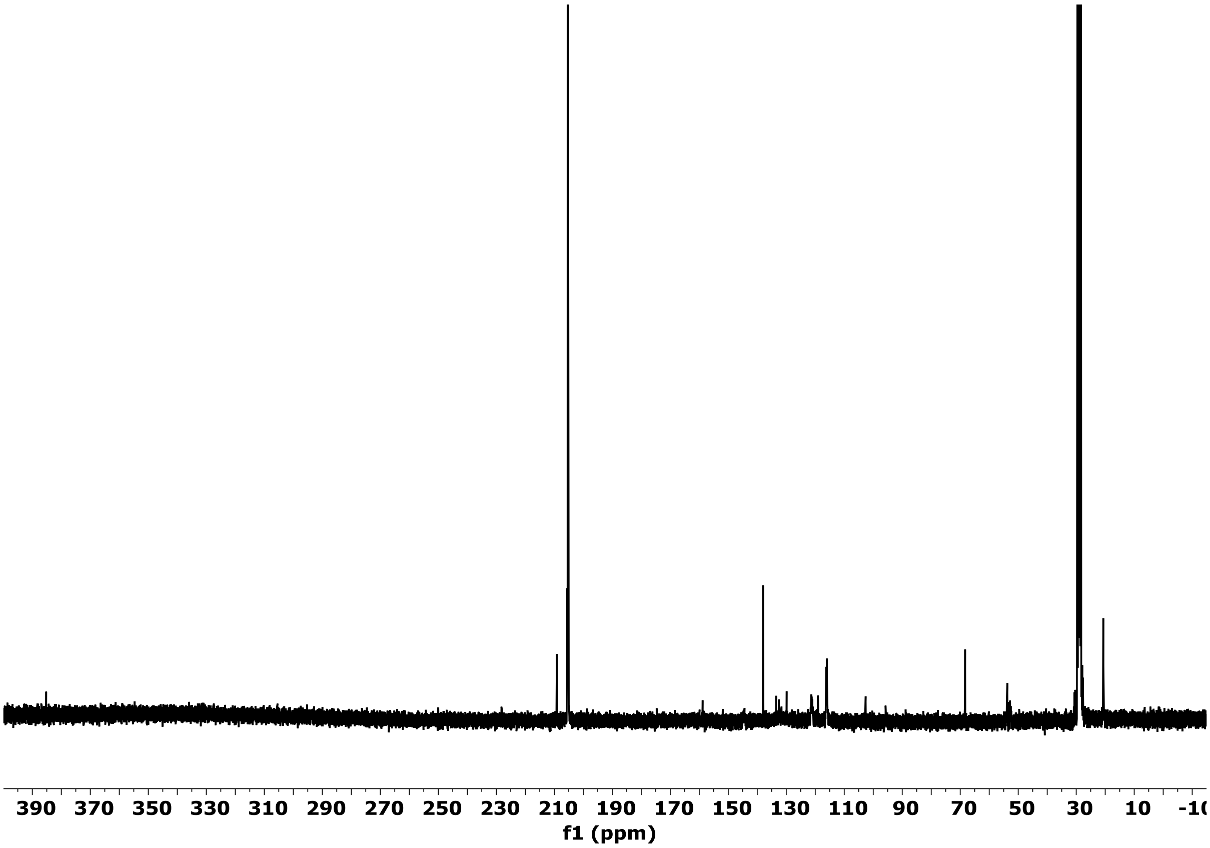

Supplementary Fig. 5. ^{13}C NMR spectrum of **VIII-d** in deuterated acetone.

4. Studies on the solvent effect

Supplementary Table 1. Alkyne metathesis in different solvents using catalyst **VIII-d**.

Solvent	Yield (%)	Solvent	Yield (%)
CCl_4	96	Acetone	< 1
$CHCl_3$	79	Toluene	18
Dichloroethane	4	CH_3CN	< 1
THF	1	Chlorobenzene	76

Reaction conditions: The ligand **1d** (1.1 mg, 0.003 mmol) and the precursor (2.0 mg, 0.003 mmol) were mixed in a dry solvent (2 mL) and stirred for 10 minutes at 70 ºC to generate the catalyst *in situ.* To the generated catalyst solution was added a solution of 4-propynylbenzlaldehyde (14.4 mg, 0.1 mmol) in a dry solvent (1 mL) and 5 Å molecular sieves (150 mg). The resulting suspension was heated at 70 ºC for 30 min under argon. The yields were estimated based on the proton signal integrations in the crude product ^{1}H NMR spectra.

Supplementary Table 2. Alkyne metathesis in co-solvents (v/v, CCl_4 /Solvent = 1/9) using catalyst **VIII-d**.

Solvent	Yield (%)	Solvent	Yield (%)
CCl_4	96	Acetone	8
$CHCl_3$	80	Toluene	90
Dichloroethane	37	CH_3CN	4
THF	18	Chlorobenzene	91

Reaction conditions: The ligand **1d** (1.1 mg, 0.003 mmol) and the precursor (2.0 mg, 0.003 mmol) were mixed in dry carbon tetrachloride (0.3 mL) and stirred for 10 minutes at 70 ºC to generate the catalyst *in situ.* To the generated catalyst solution was added a solution of 4-propynylbenzlaldehyde (14.4 mg, 0.1 mmol) in a chosen solvent (2.7 mL) and 5 Å molecular sieves (150 mg). The resulting suspension was heated at 70 ºC for 30 min under argon. The yields were estimated based on the proton signal integrations in the crude product ^{1}H NMR spectra.

5. Kinetic study of the alkyne metathesis catalyzed by the catalyst VIII-d

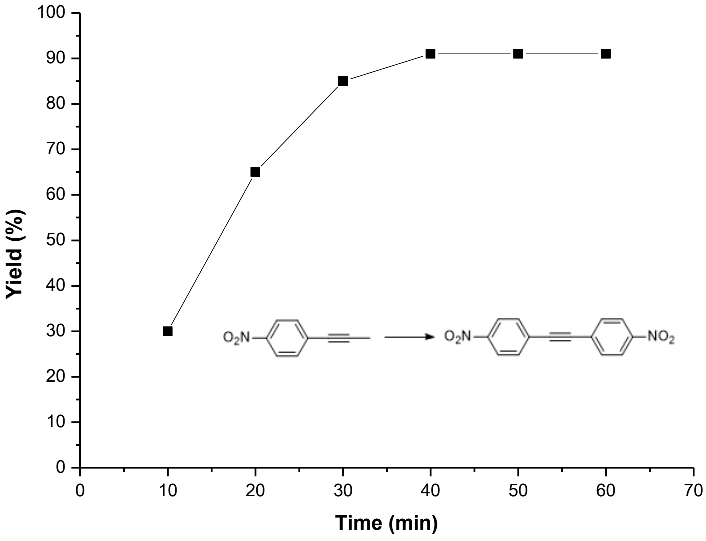

Supplementary Fig. 6. Kinetic study of the alkyne metathesis of 1-nitro-4-propynylbenzene.

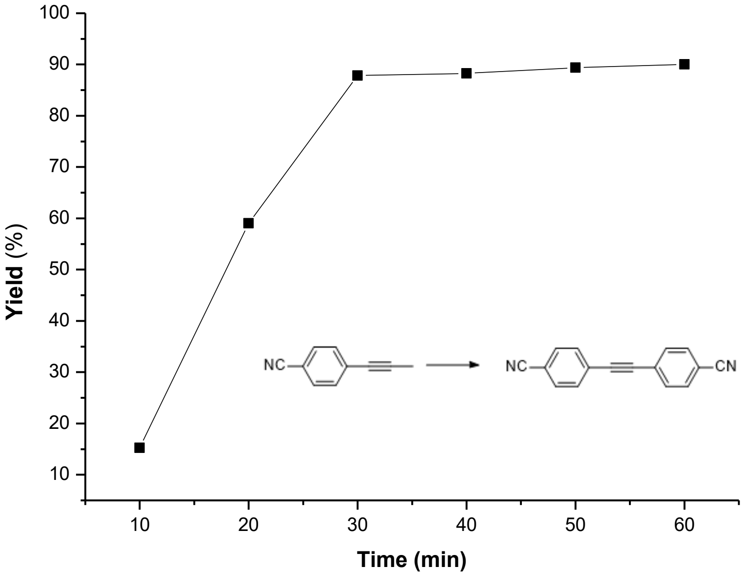

Supplementary Fig. 7. Kinetic study of the alkyne metathesis of 1-cyano-4-propynylbenzene.

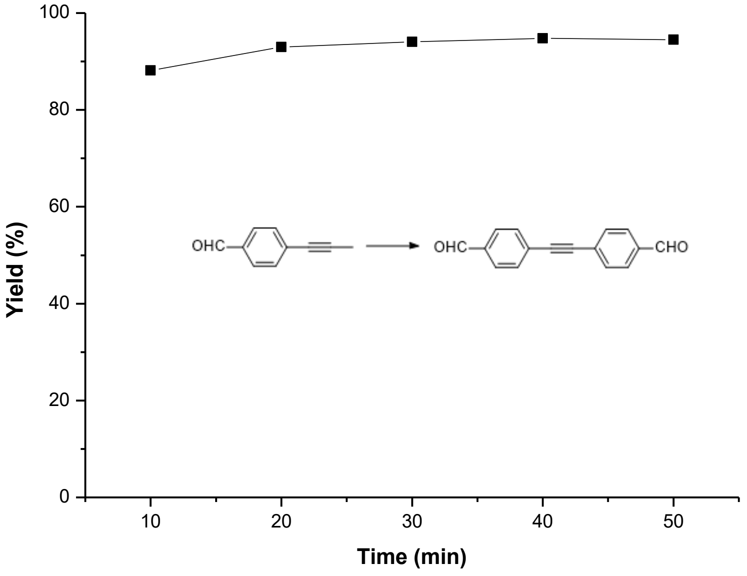

Supplementary Fig. 8. Kinetic study of the alkyne metathesis of 1-formyl-4-propynylbenzene.

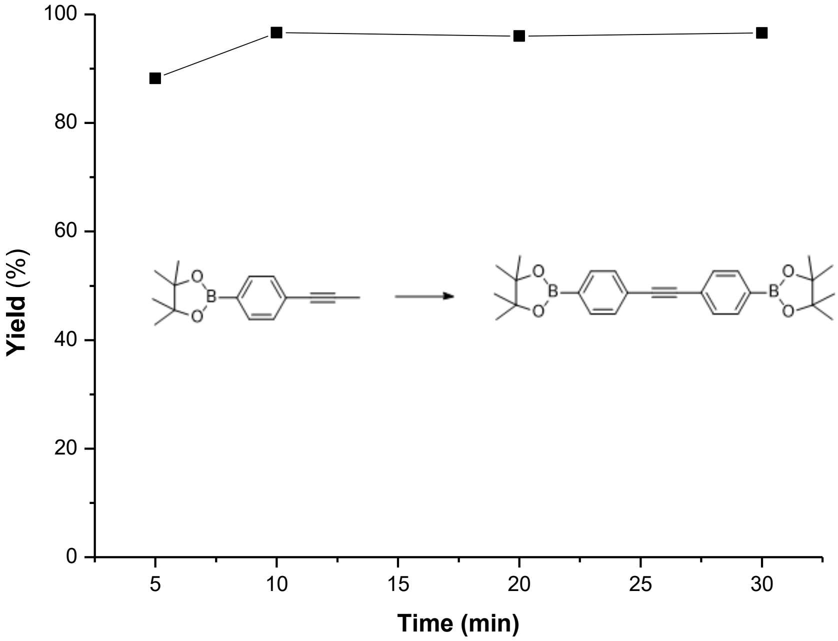

Supplementary Fig. 9. Kinetic study of the alkyne metathesis of 1-borate ester-4-propynylbenzene.

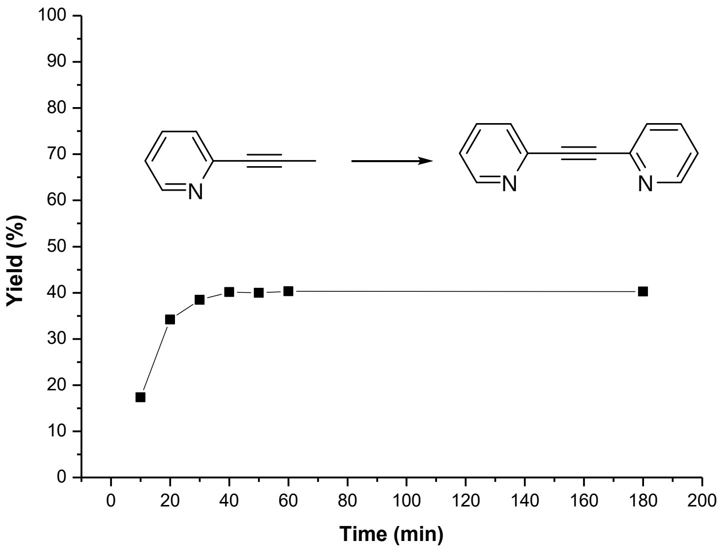

Supplementary Fig. 10. Kinetic study of the alkyne metathesis of 2-(prop-1-yn-1-yl) pyridine.

6. Catalytic activity comparison between catalysts VIII-d and VIId (R^1 = ^{i}Pr, R^2 = H)

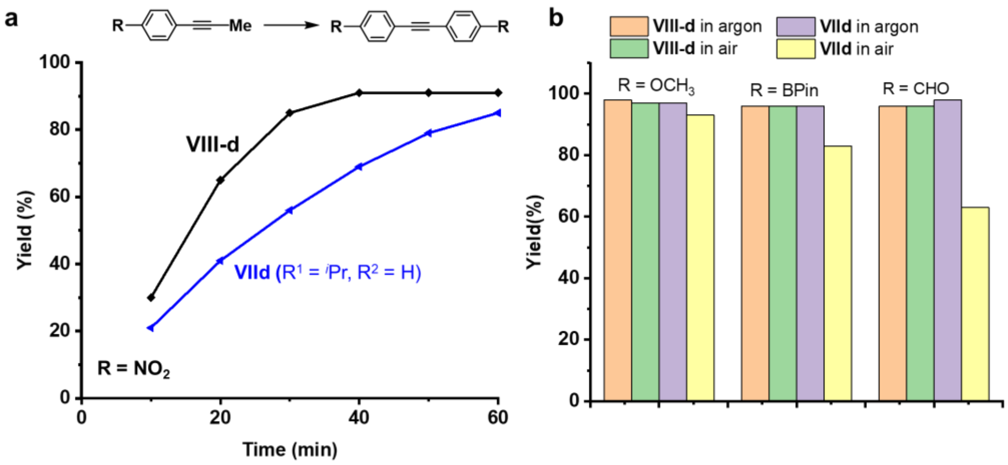

Supplementary Fig. 11. Catalytic activity comparison of **VIII-d** and **VIId** (R^1 = ^{i}Pr, R^2 = H): (a) Comparison of the kinetic profiles of the metathesis of 1-nitro-4-propynyl benzene under argon. Conditions: 0.1 mmol substrate, 150 mg MS 5Å, and 3.0 mL CCl_4, 0.003 mmol precursor, and 0.003 mmol ligand were used. (b) Comparison of the catalytic activities of **VIII-d** and **VIId** in air and argon. Conditions for reactions in open air: 0.1 mmol substrate, 0.003 mmol catalyst (3 mol%), 3 mL CCl_4, 70 °C, 30 min; Conditions for reactions under argon: 0.1 mmol substrate, 0.003 mmol catalyst (3 mol%), 3 mL CCl_4, 150 mg 5Å MS, 70 °C, 30 min.

7. Alkyne metathesis with different alkynes

Supplementary Table 3. Alkyne metathesis of challenging substrates

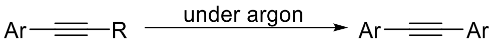

Entry	Substrate	Product	Yield (%)
1	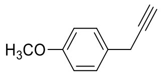	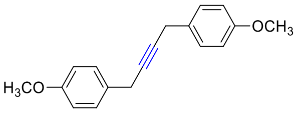	0
2	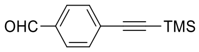	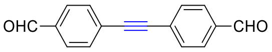	3
3	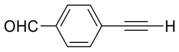	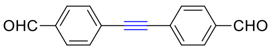	0

Conditions: The ligand **VIII-1d** (1.1 mg, 0.003 mmol) and the precursor (2.0 mg, 0.003 mmol) were mixed in dry carbon tetrachloride (2 mL) and stirred for 10 minutes at 70 ºC to generate the catalyst *in situ*. To the generated catalyst solution was added the solution of the substrate (0.1 mmol) in CCl_4 (1 mL) and 5 Å molecular sieves (150 mg). The resulting suspension was heated at 70 ºC for 30 min under Ar. The yields were estimated based on the proton signal integrations in the crude product ^{1}H NMR spectra.

8. Computational calculations

Computations were performed by the Gaussian09 suites of programs in this work. Molecular geometries were optimized at the B3LYP level of density functional theory. The SDD basis set was used for Mo, and the 6-31G** basis set was used for C, N, O and H atoms.

8.1 Optimized ligand structures and energy comparison

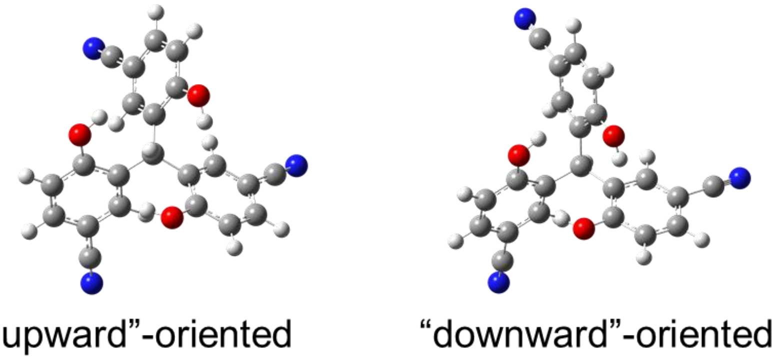

Supplementary Fig. 12. Two conformations of **1d** with the central C-H group oriented upward (the same direction with the three OH groups) or downward (opposite direction with the three OH groups).

Supplementary Table 4. The energy difference between the two conformers of the ligands **1a-d**.

Ligands	$\Delta_r G$ (kJ/mol)	$\Delta_r G$ (Kcal/mol)	$\Delta_r H$ (kJ/mol)	$\Delta_r H$ (kcal/mol)
**1a**	-15.60	-3.73	-17.74	-4.24
**1b**	-21.31	-5.10	-22.74	-5.44
**1c**	-17.24	-4.13	-18.55	-4.44
**1d**	-21.78	-5.21	-23.12	-5.53

$\Delta_r G = G_{upward} - G_{downward}$ $\Delta_r H = H_{upward} - H_{downward}$

9. ^{1}H and ^{13}C NMR spectra for selected compounds

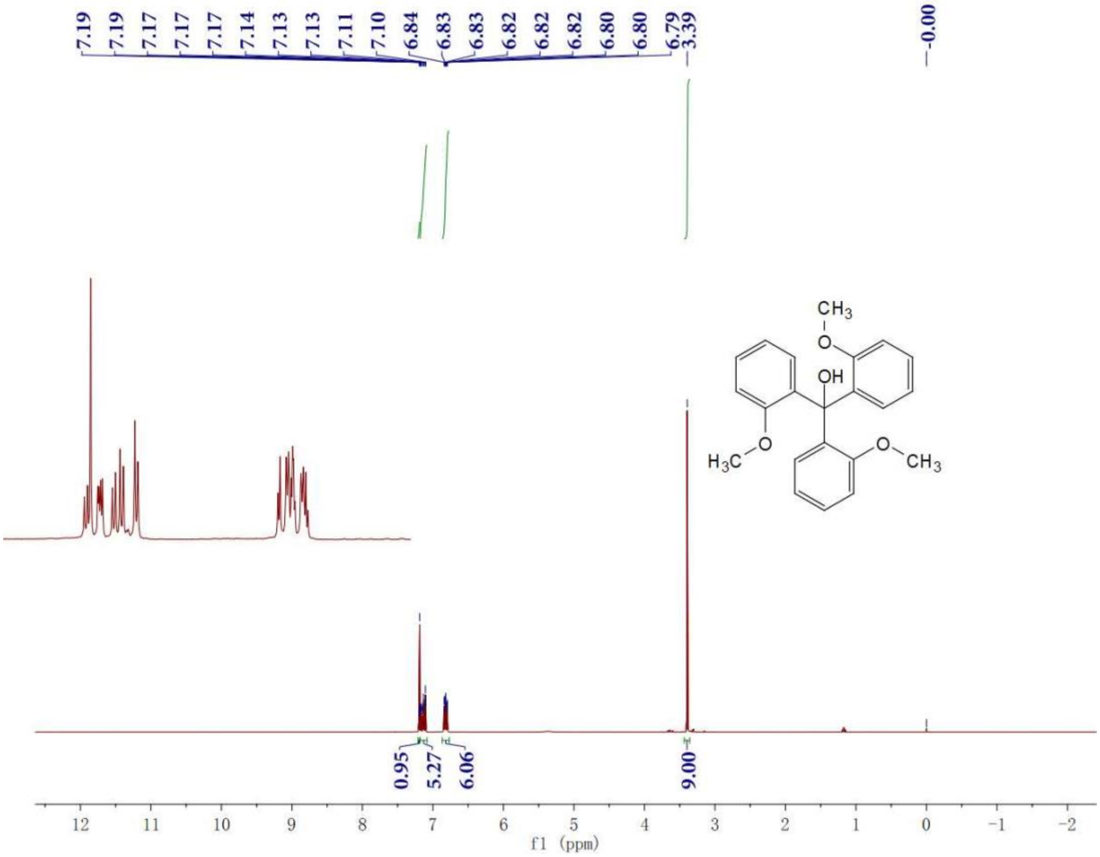

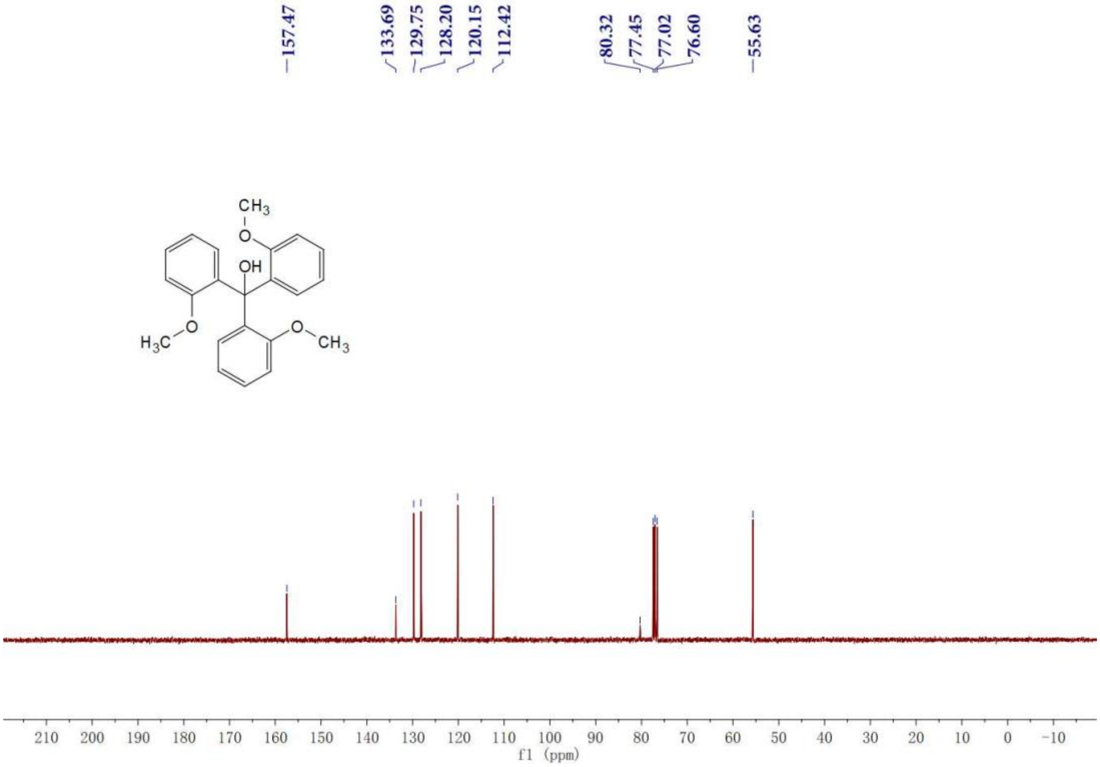

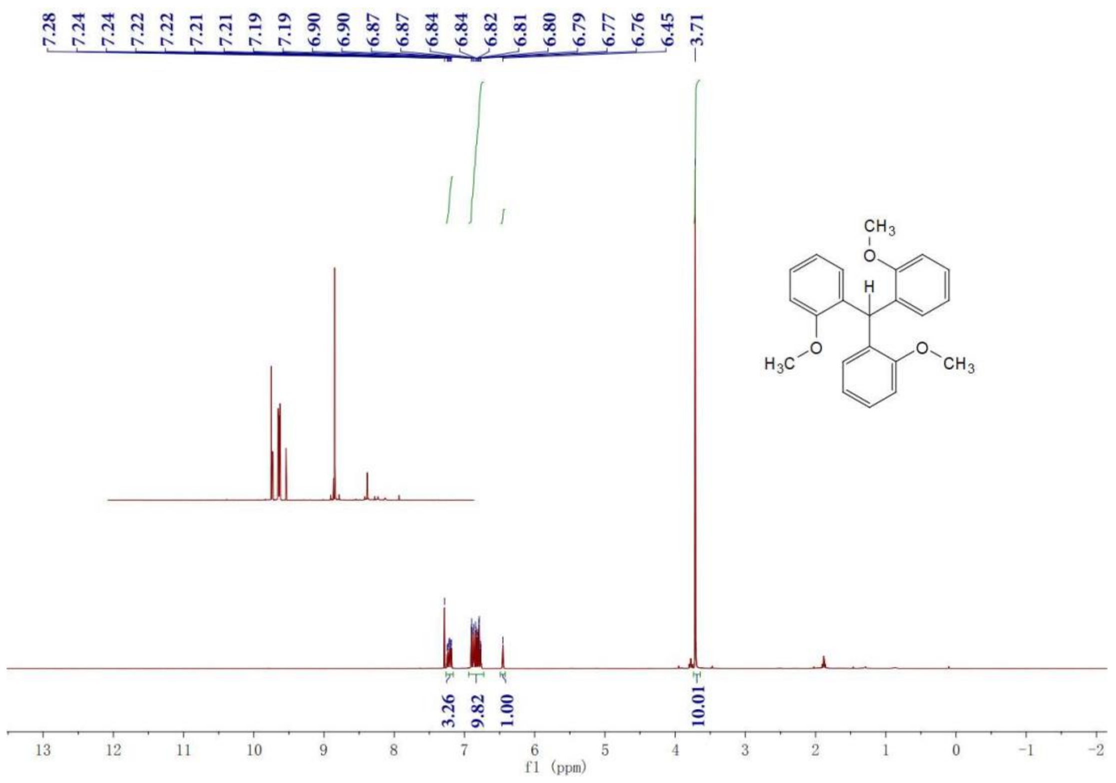
7.28 7.24 7.24 7.22 7.22 7.21 7.21 7.19 7.19 6.90 6.90 6.87 6.87 6.84 6.84 6.82 6.81 6.80 6.79 6.77 6.76 6.45
3.71
CH3
O
H
O
H3C
O
CH3
3.26 9.82 1.00
10.01
f1 (ppm)

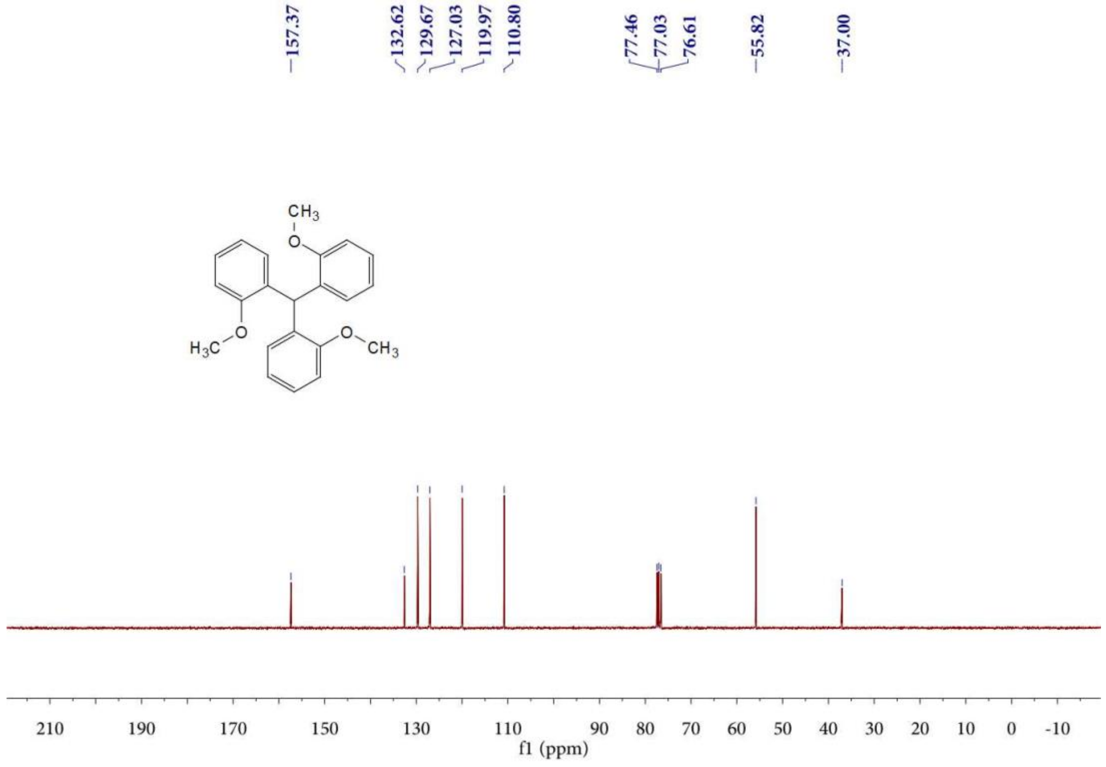
157.37
132.62 129.67 127.03 119.97 110.80
77.46 77.03 76.61
55.82
37.00
CH3
O
O
H3C
O
CH3
f1 (ppm)

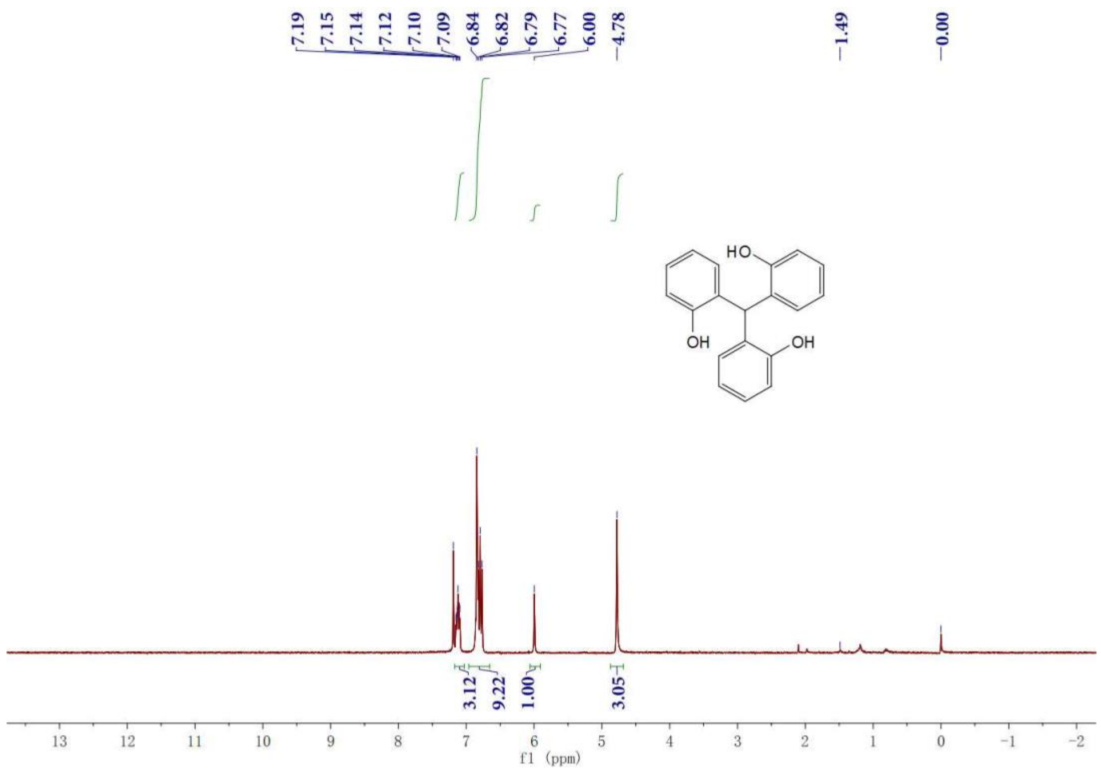
7.19
7.15
7.14
7.12
7.10
7.09
6.84
6.82
6.79
6.77
6.00
4.78
1.49
0.00
HO
OH
OH
3.12
9.22
1.00
3.05
f1 (ppm)

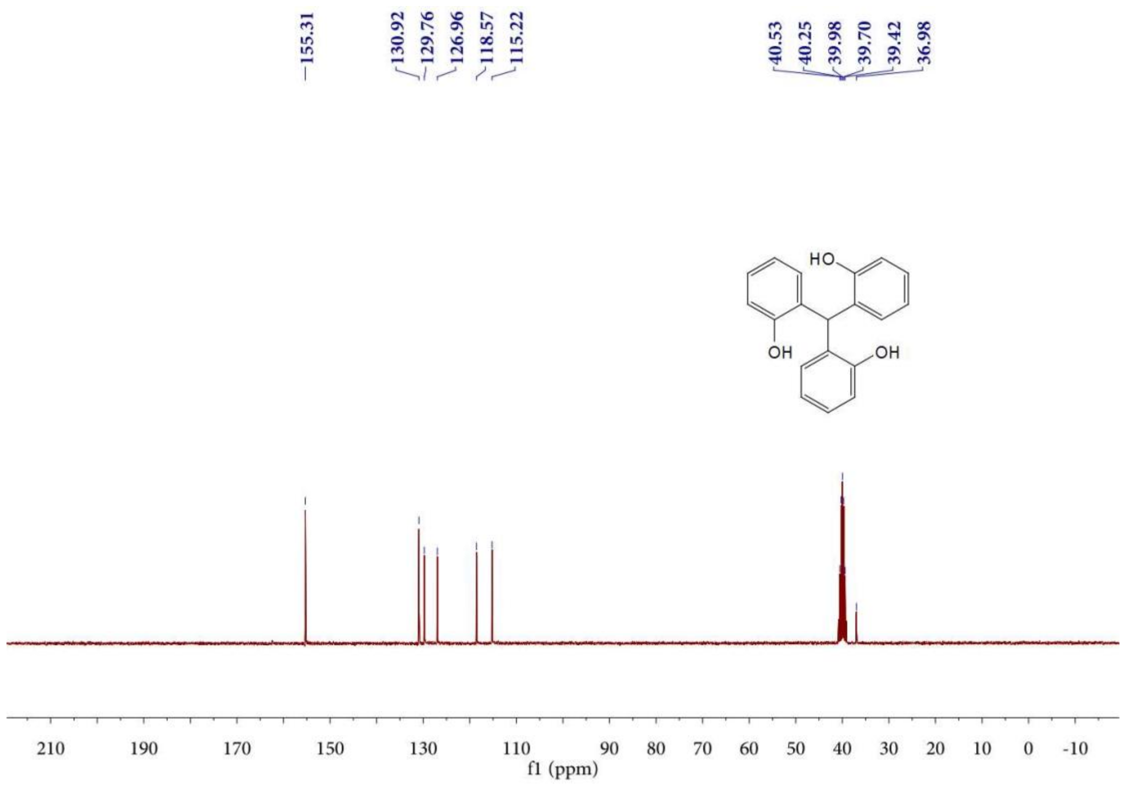
155.31
130.92
129.76
126.96
118.57
115.22
40.53
40.25
39.98
39.70
39.42
36.98
HO
OH
OH
f1 (ppm)

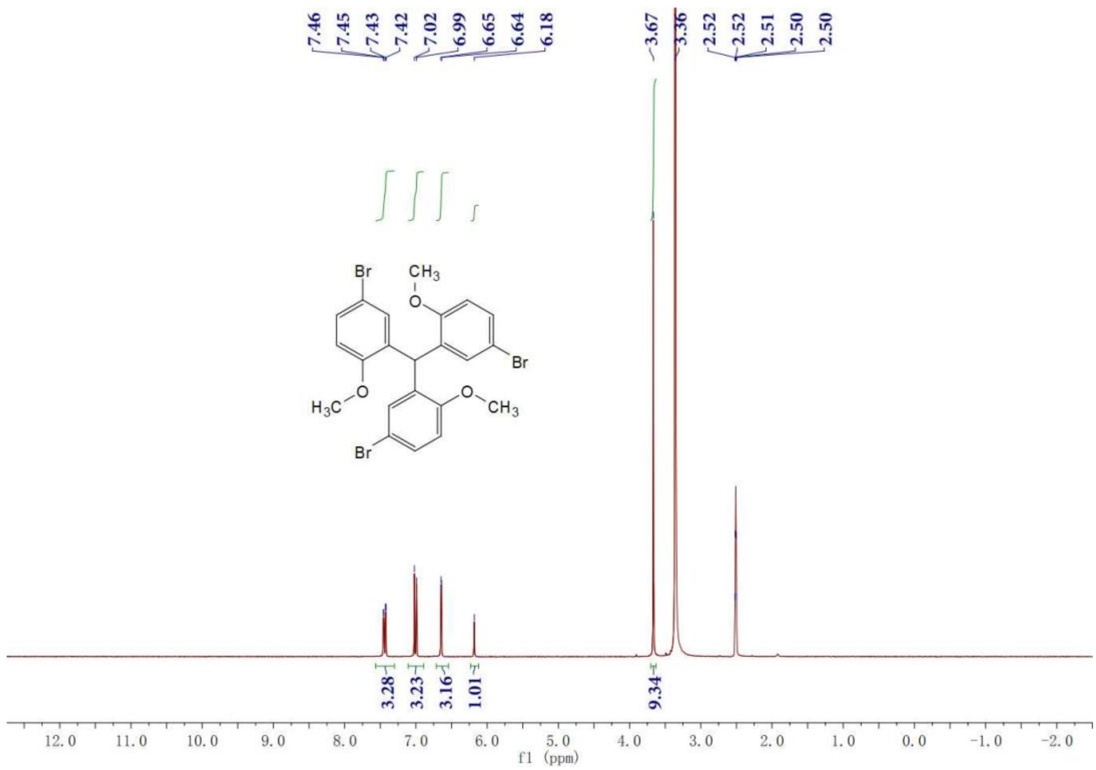
7.46
7.45
7.43
7.42
7.02
6.99
6.65
6.64
6.18
3.67
3.36
2.52
2.52
2.51
2.50
2.50
Br
CH_3
O
Br
H_3C
O
O
CH_3
Br
3.28
3.23
3.16
1.01
9.34
12.0 11.0 10.0 9.0 8.0 7.0 6.0 5.0 4.0 3.0 2.0 1.0 0.0 -1.0 -2.0
f1 (ppm)

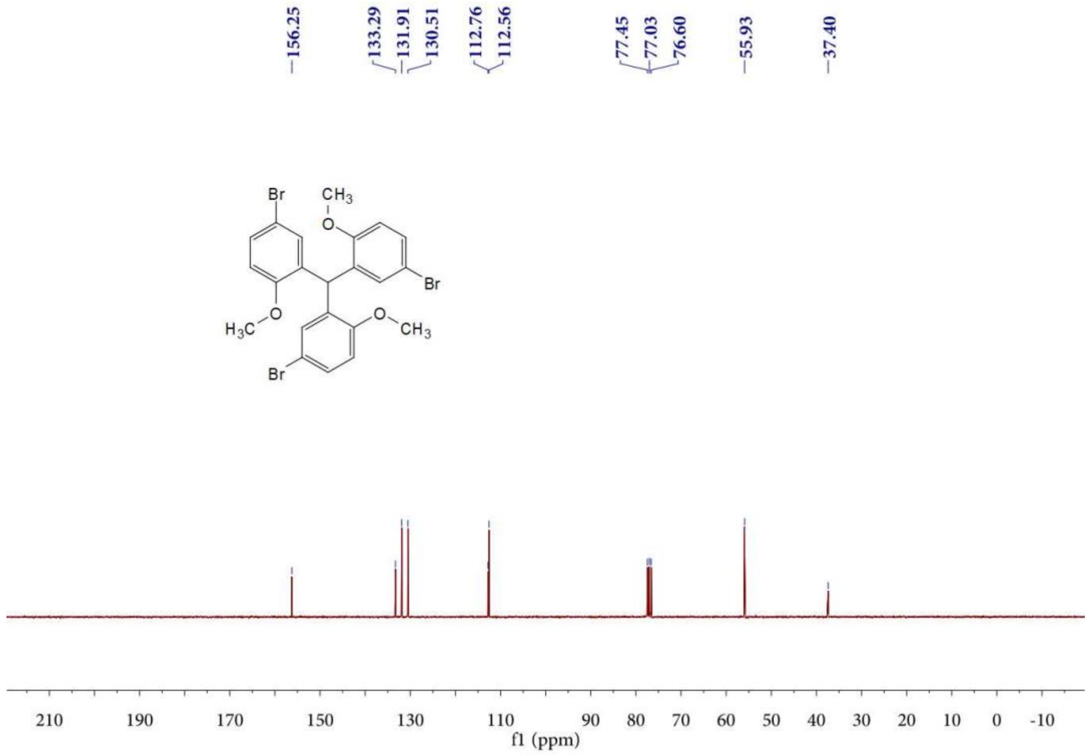
156.25
133.29
131.91
130.51
112.76
112.56
77.45
77.03
76.60
55.93
37.40
Br
CH_3
O
Br
H_3C
O
O
CH_3
Br
210 190 170 150 130 110 90 80 70 60 50 40 30 20 10 0 -10
f1 (ppm)

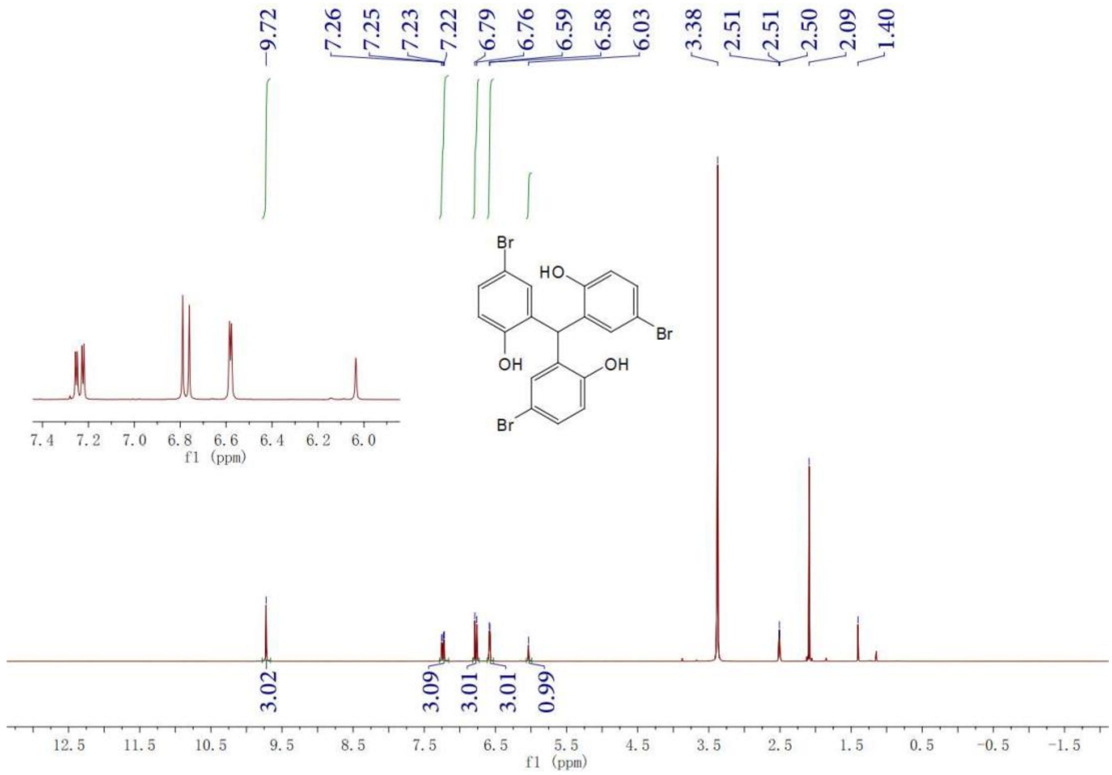
9.72
7.26 7.25 7.23 7.22 6.79 6.76 6.59 6.58 6.03 3.38 2.51 2.51 2.50 2.09 1.40
Br
HO
Br
OH
OH
Br
3.02
3.09
3.01
3.01
0.99
f1 (ppm)

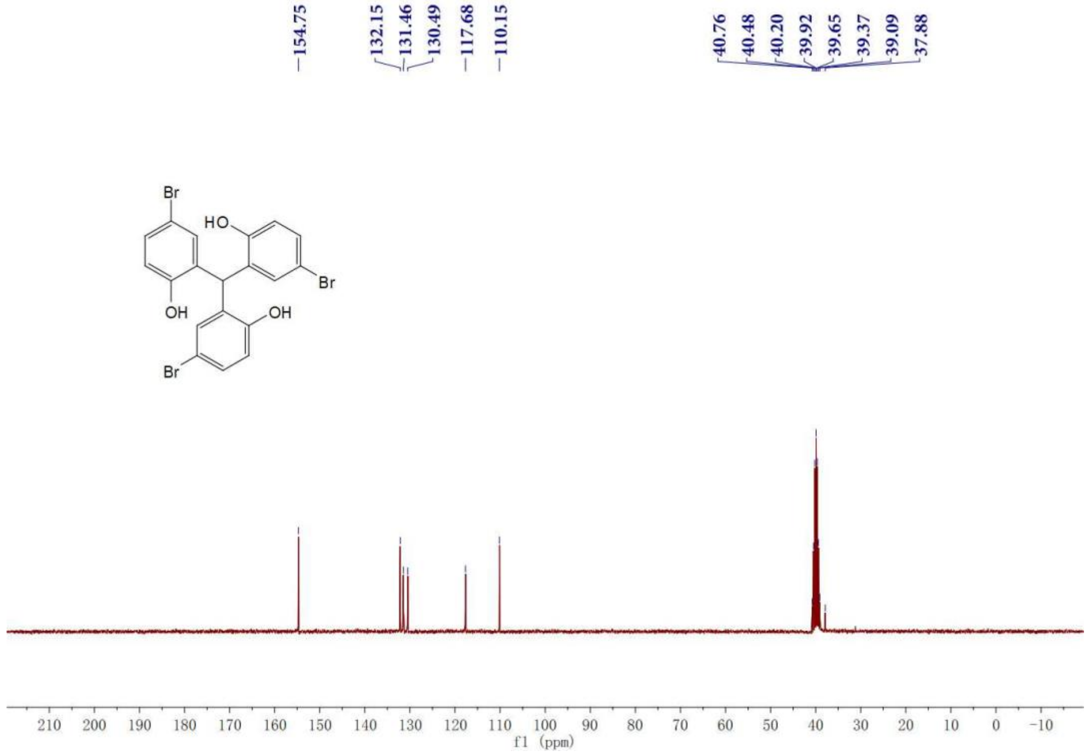
154.75
132.15 131.46 130.49 117.68 110.15
40.76 40.48 40.20 39.92 39.65 39.37 39.09 37.88
Br
HO
Br
OH
OH
Br
f1 (ppm)

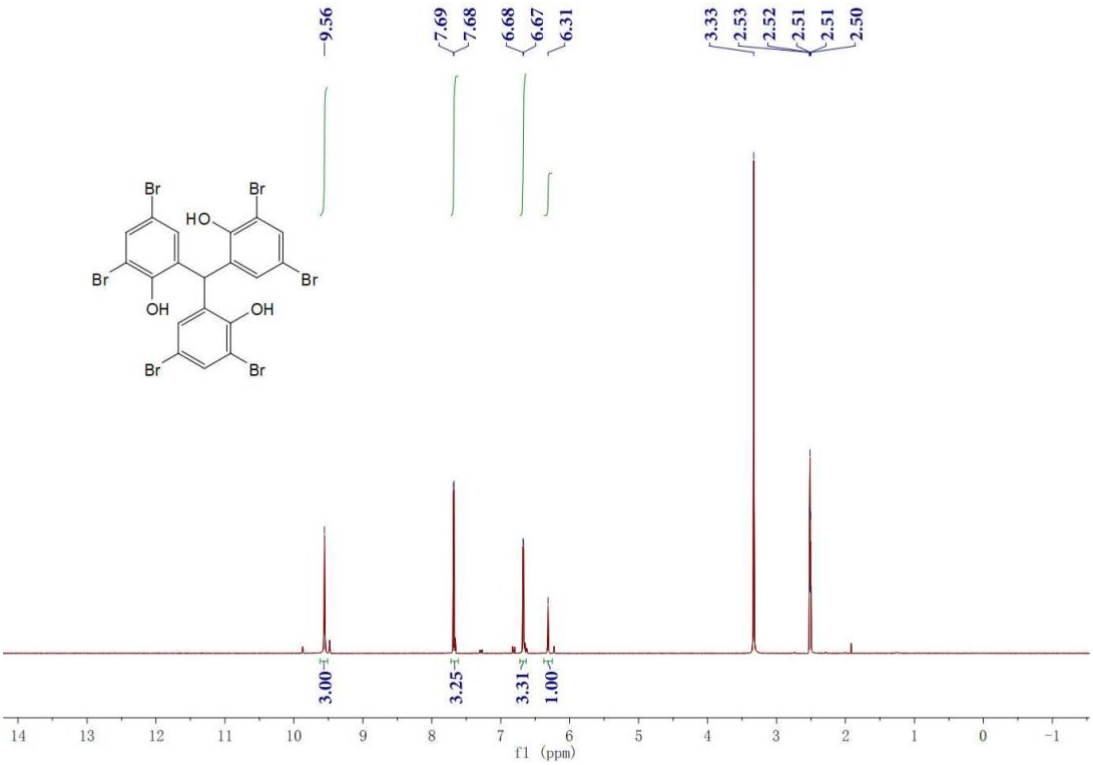
9.56
7.69
7.68
6.68
6.67
6.31
3.33
2.53
2.52
2.51
2.51
2.50
Br
HO
Br
Br
Br
OH
OH
Br
Br
3.00
3.25
3.31
1.00
14 13 12 11 10 9 8 7 6 5 4 3 2 1 0 -1
f1 (ppm)

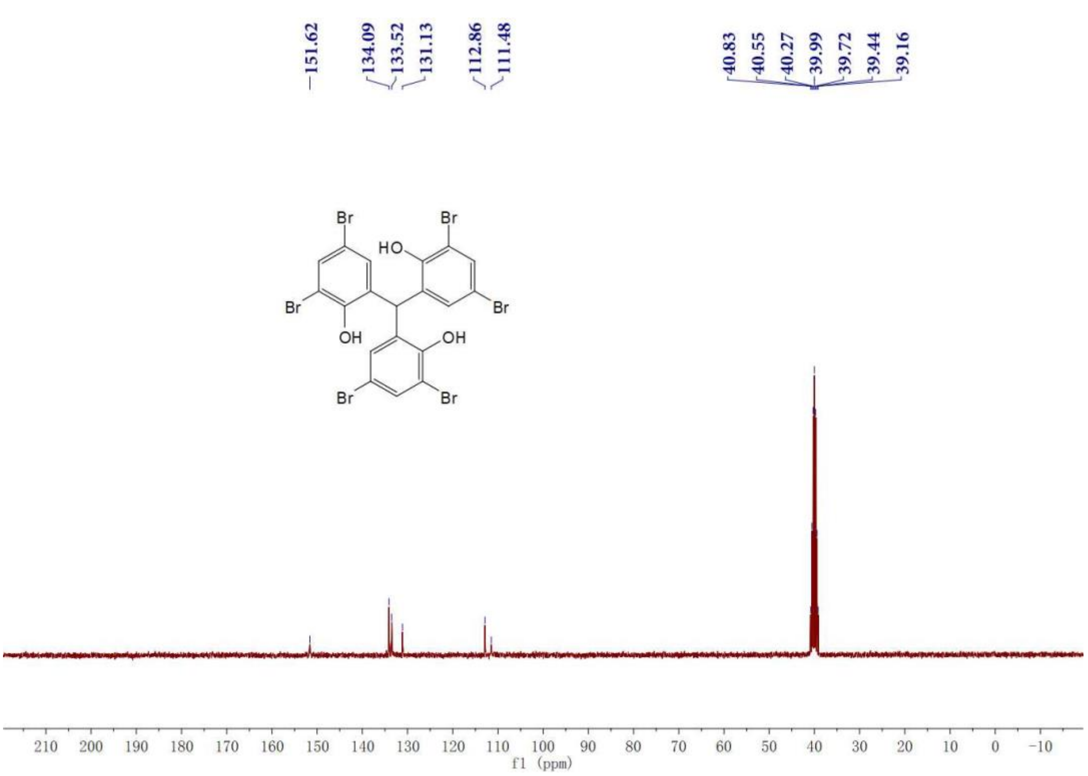
151.62
134.09
133.52
131.13
112.86
111.48
40.83
40.55
40.27
39.99
39.72
39.44
39.16
Br
HO
Br
Br
Br
OH
OH
Br
Br
210 200 190 180 170 160 150 140 130 120 110 100 90 80 70 60 50 40 30 20 10 0 -10
f1 (ppm)

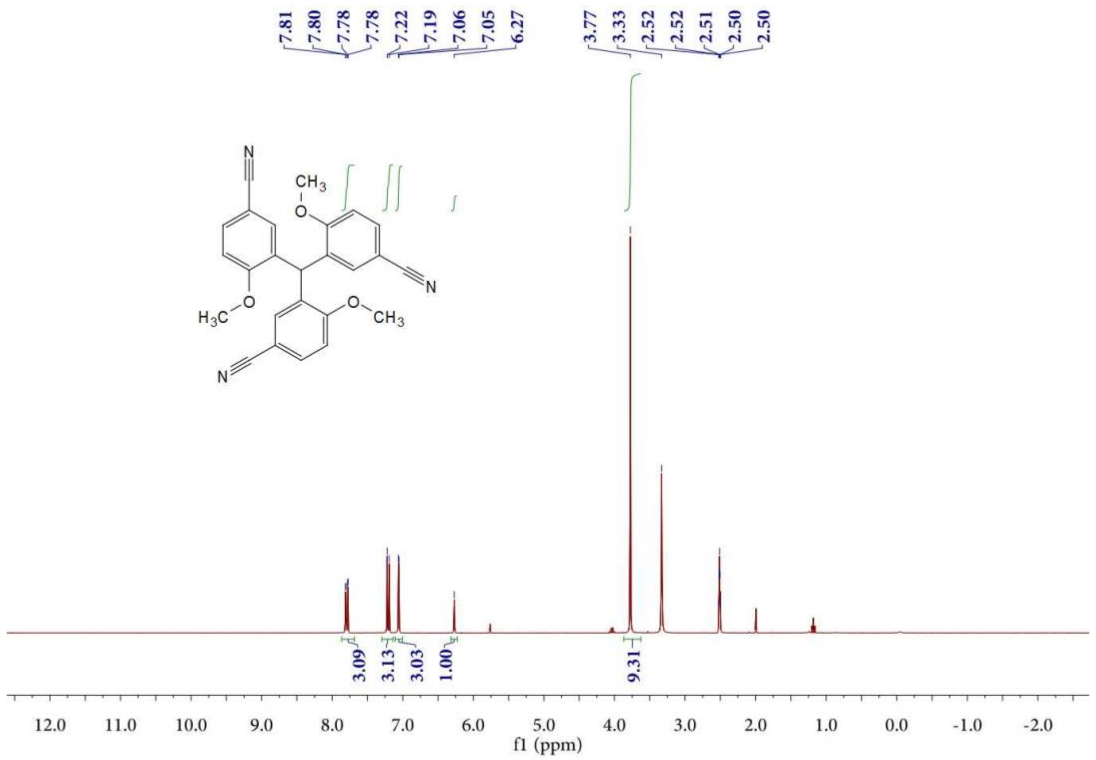
7.81 7.80 7.78 7.78 7.22 7.19 7.06 7.05 6.27
3.77 3.33 2.52 2.52 2.51 2.50 2.50
3.09 3.13 3.03 1.00 9.31
12.0 11.0 10.0 9.0 8.0 7.0 6.0 5.0 4.0 3.0 2.0 1.0 0.0 -1.0 -2.0
f1 (ppm)

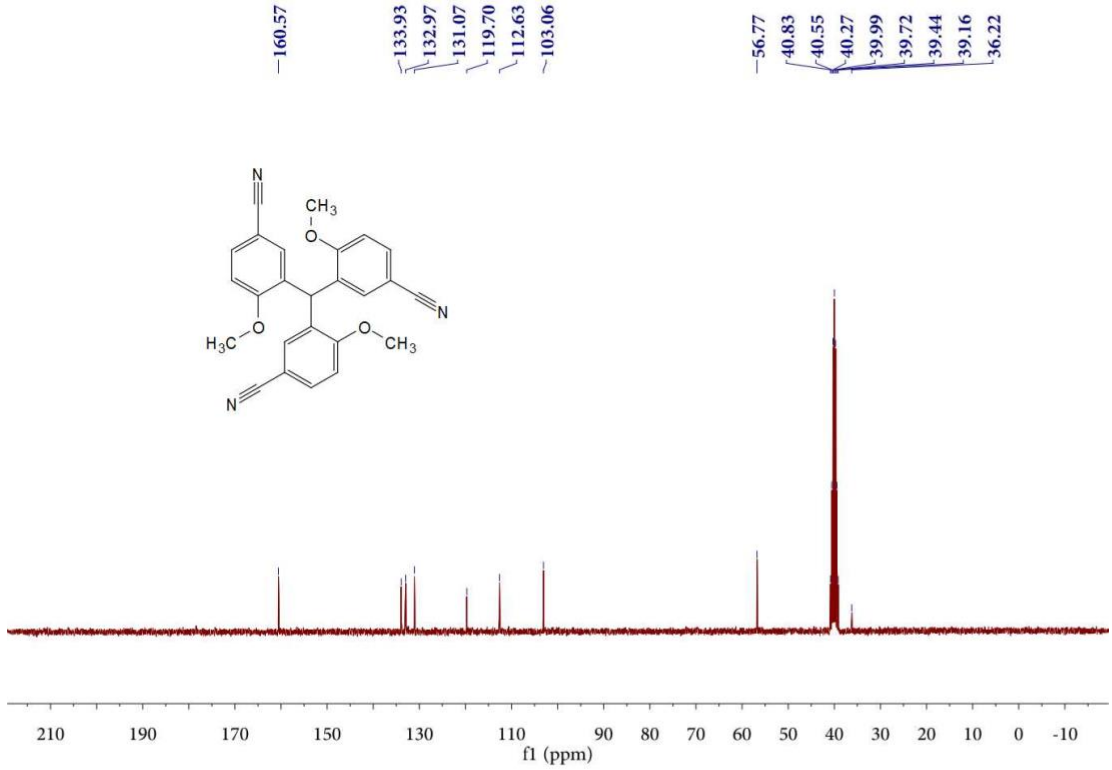
160.57
133.93 132.97 131.07 119.70 112.63 103.06
56.77 40.83 40.55 40.27 39.99 39.72 39.44 39.16 36.22
210 190 170 150 130 110 90 80 70 60 50 40 30 20 10 0 -10
f1 (ppm)

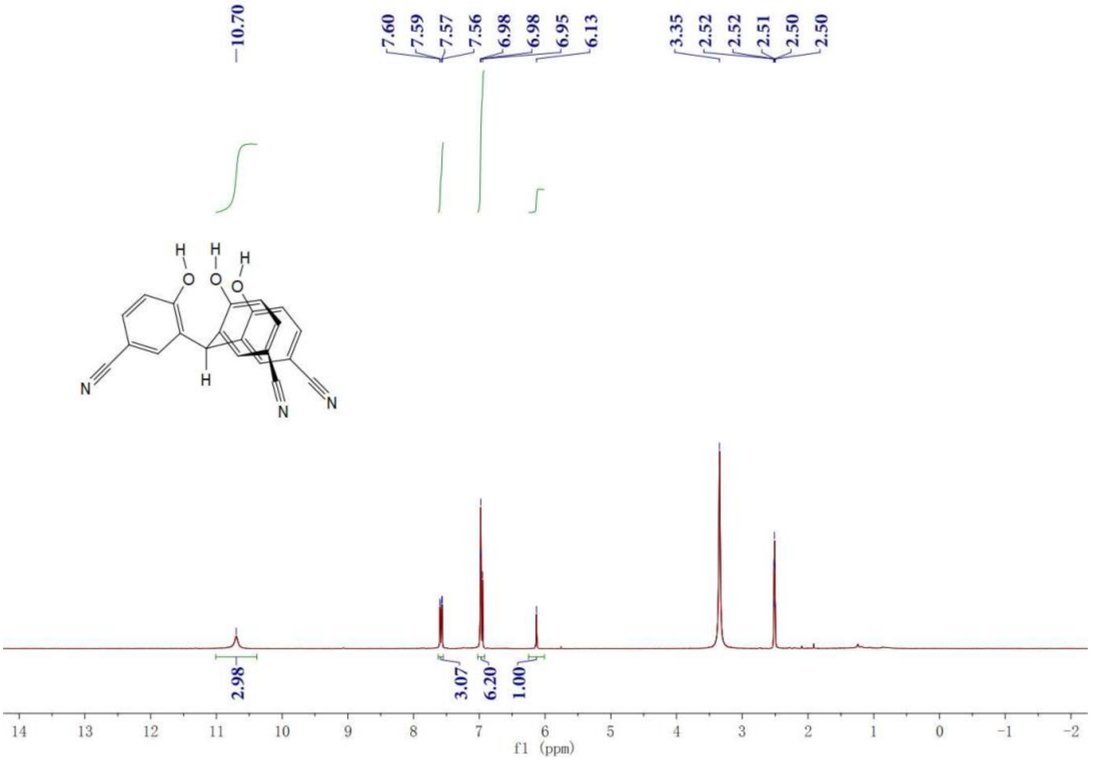

10.70
7.60
7.59
7.57
7.56
6.98
6.98
6.95
6.13
3.35
2.52
2.52
2.51
2.50
2.50
2.98
3.07
6.20
1.00
f1 (ppm)

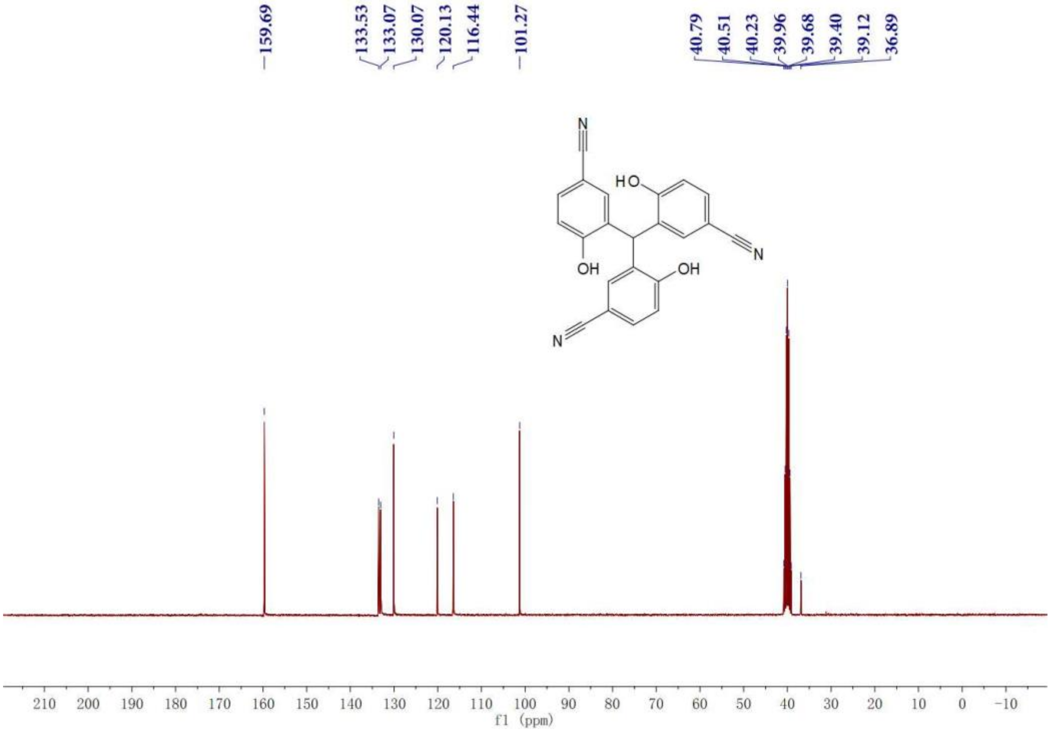

159.69
133.53
133.07
130.07
120.13
116.44
101.27
40.79
40.51
40.23
39.96
39.68
39.40
39.12
36.89
f1 (ppm)

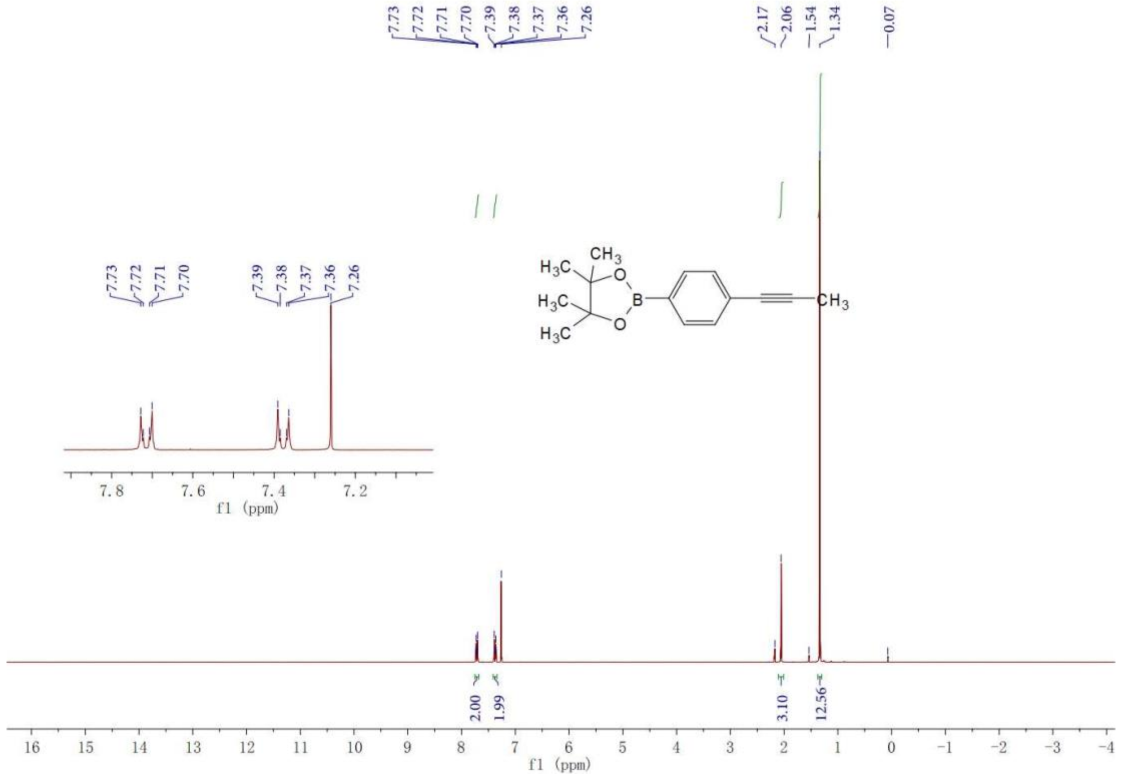
7.73 7.72 7.71 7.70 7.39 7.38 7.37 7.36 7.26
2.17 2.06 1.54 1.34
0.07
2.00
1.99
3.10
12.56
f1 (ppm)

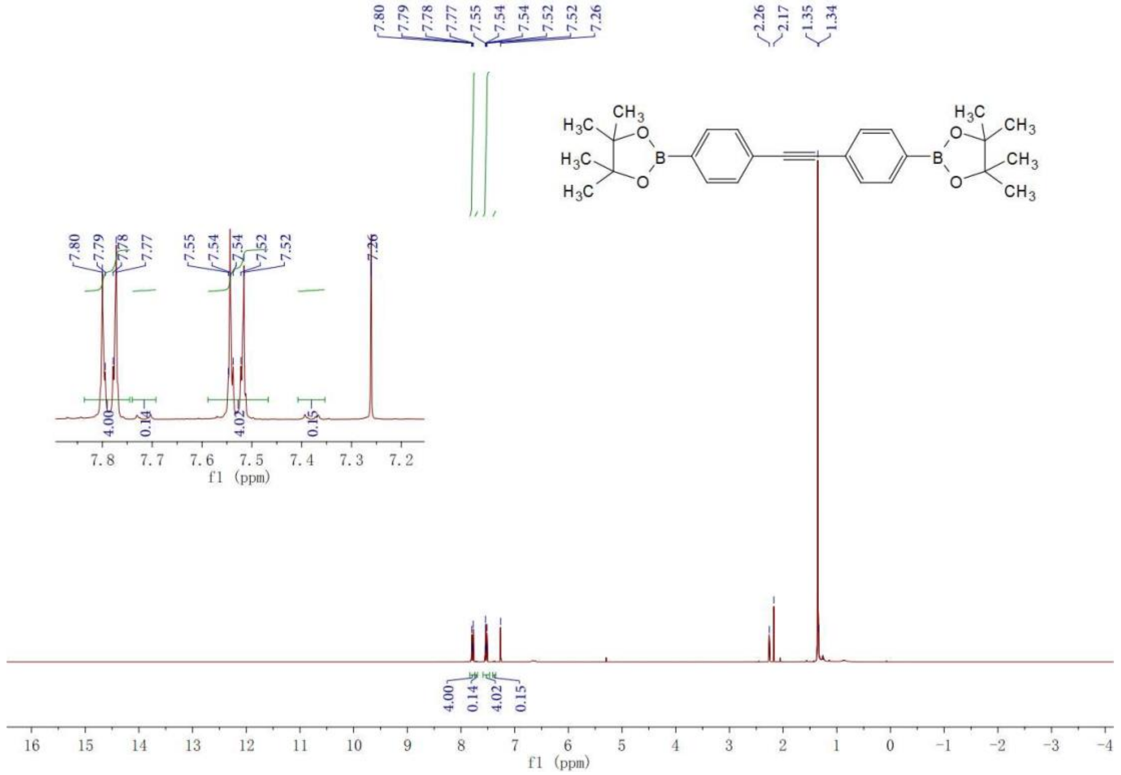
7.80 7.79 7.78 7.77 7.55 7.54 7.54 7.52 7.52 7.26
2.26 2.17 1.35 1.34
4.00
0.14
4.02
0.15
f1 (ppm)

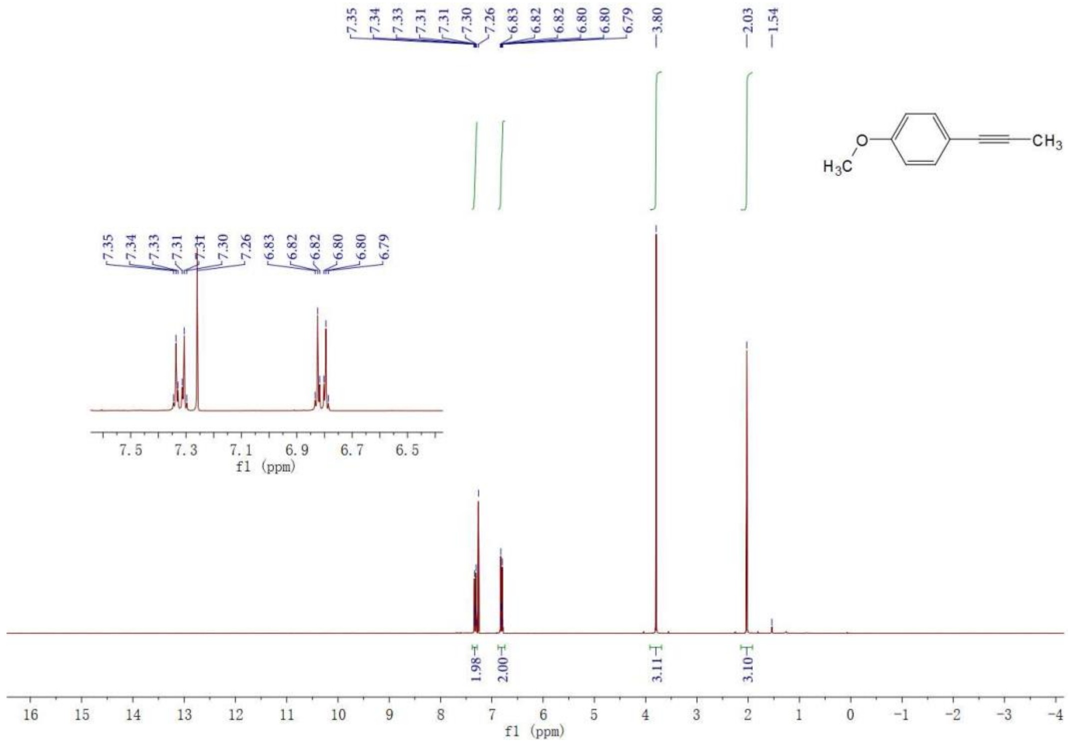
7.35 7.34 7.33 7.31 7.31 7.30 7.26 6.83 6.82 6.82 6.80 6.80 6.79
3.80
2.03
1.54
H_3C O CH3
1.98
2.00
3.11
3.10
f1 (ppm)

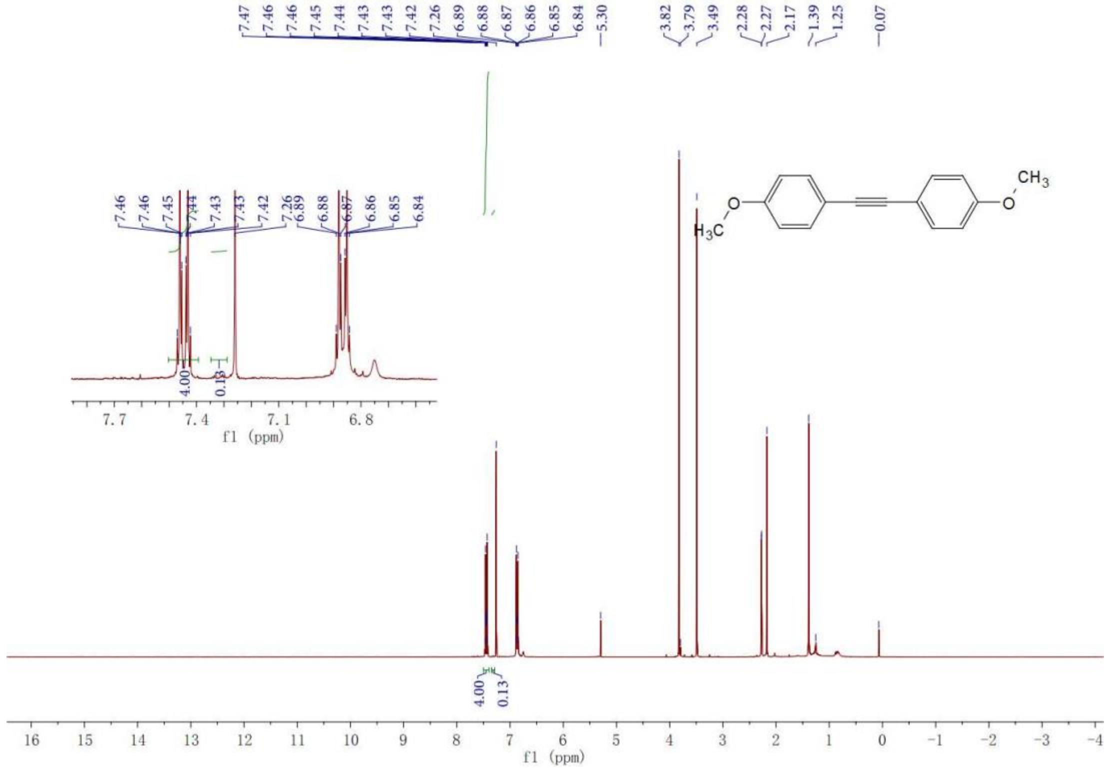
7.47 7.46 7.46 7.45 7.44 7.43 7.43 7.42 7.26 6.89 6.88 6.87 6.86 6.85 6.84
5.30
3.82 3.79 3.49
2.28 2.27 2.17 1.39 1.25
0.07
H_3C O CH_3
4.00
0.13
f1 (ppm)

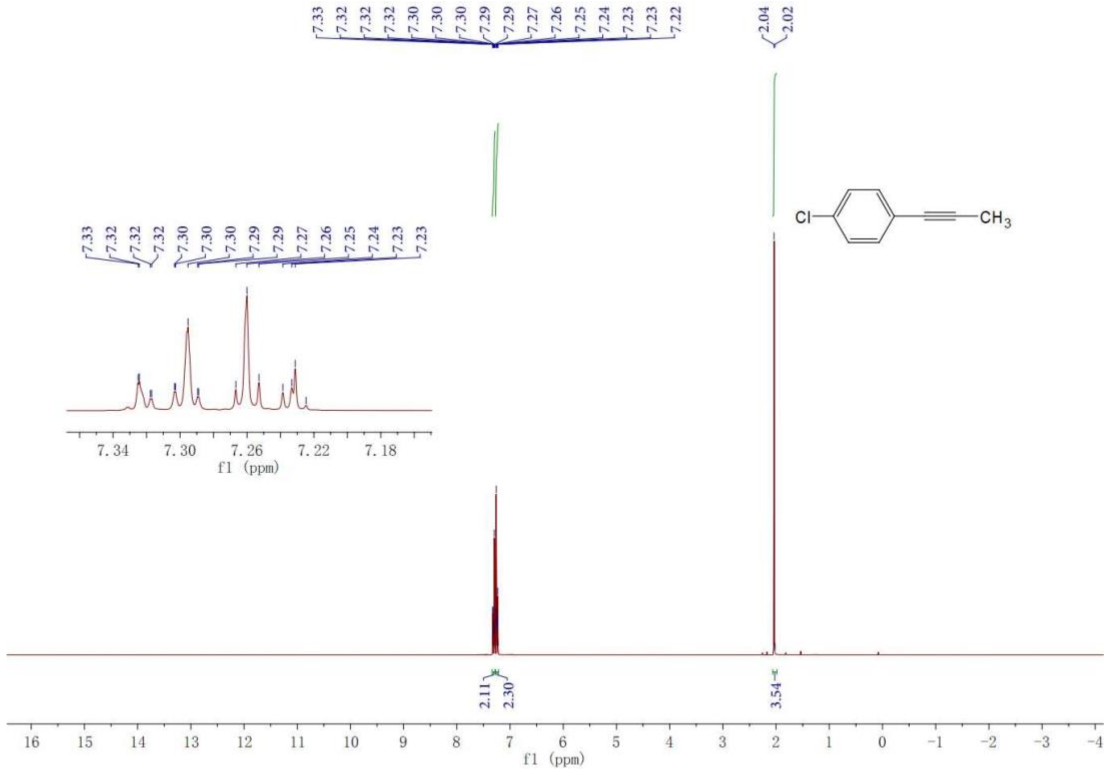
7.33 7.32 7.32 7.32 7.30 7.30 7.30 7.29 7.29 7.27 7.26 7.25 7.24 7.23 7.23 7.22
2.04 2.02
Cl
CH3
7.33 7.32 7.32 7.32 7.30 7.30 7.30 7.29 7.29 7.27 7.26 7.25 7.24 7.23 7.23
7.34 7.30 7.26 7.22 7.18
f1 (ppm)
2.11 2.30
3.54
16 15 14 13 12 11 10 9 8 7 6 5 4 3 2 1 0 -1 -2 -3 -4
f1 (ppm)

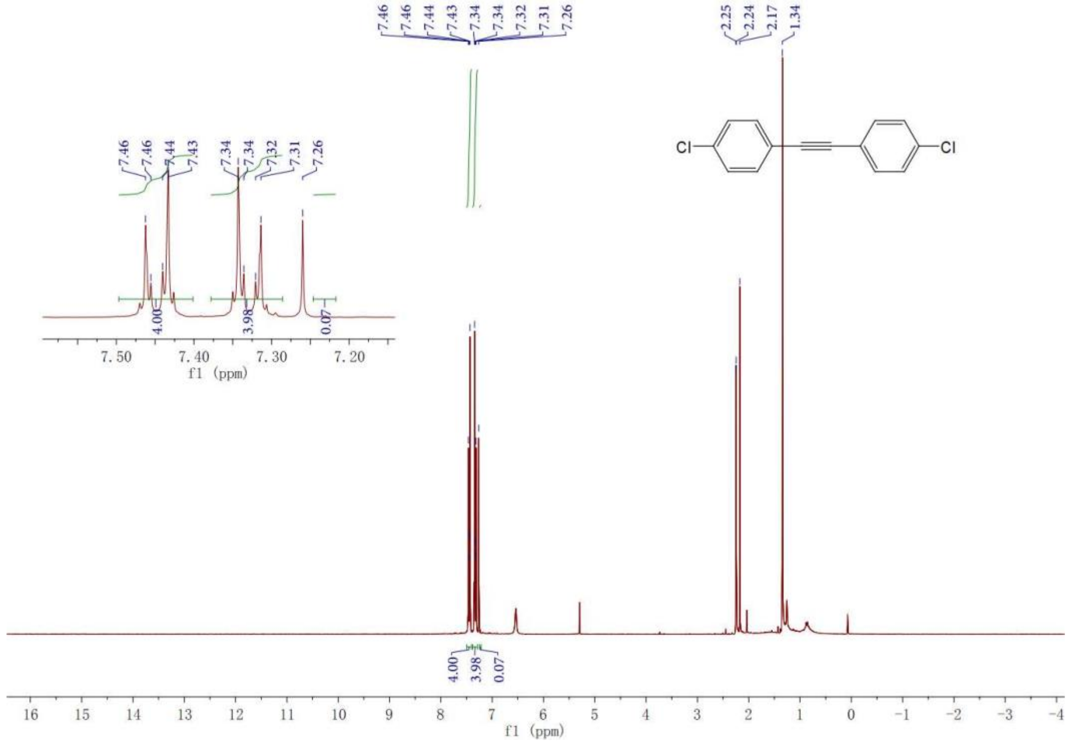
7.46 7.46 7.44 7.43 7.34 7.34 7.32 7.31 7.26
2.25 2.24 2.17 1.34
Cl
Cl
7.46 7.46 7.44 7.43 7.34 7.34 7.32 7.31 7.26
4.00 3.98 0.07
7.50 7.40 7.30 7.20
f1 (ppm)
4.00 3.98 0.07
16 15 14 13 12 11 10 9 8 7 6 5 4 3 2 1 0 -1 -2 -3 -4
f1 (ppm)

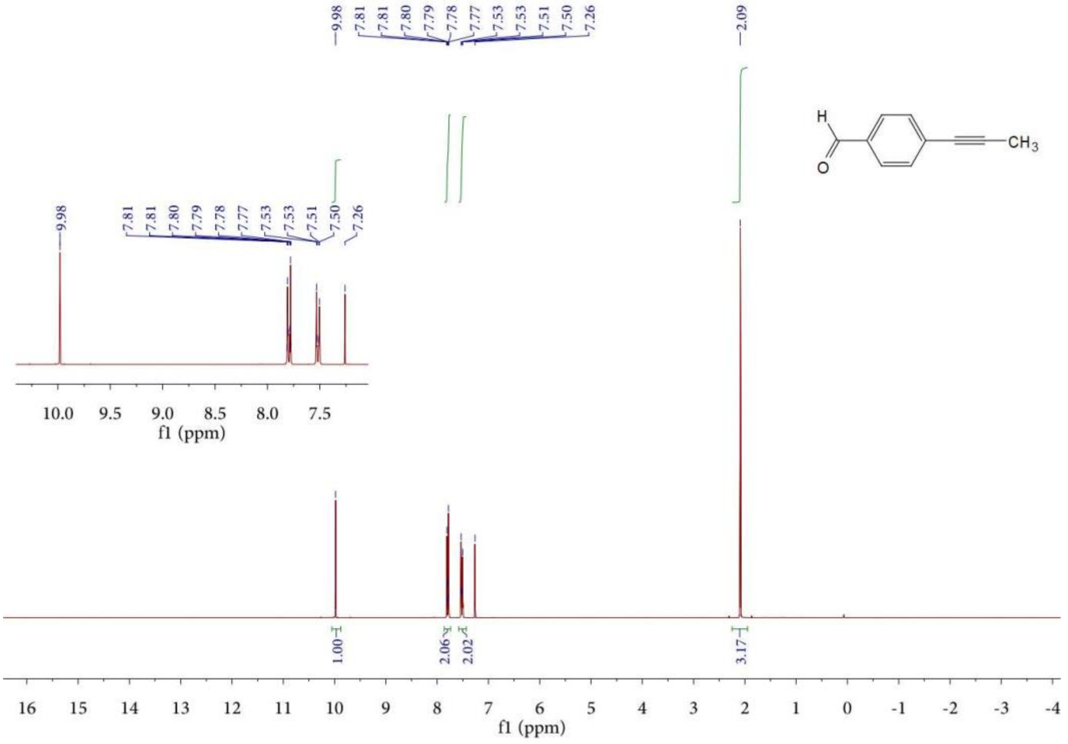
f1 (ppm)

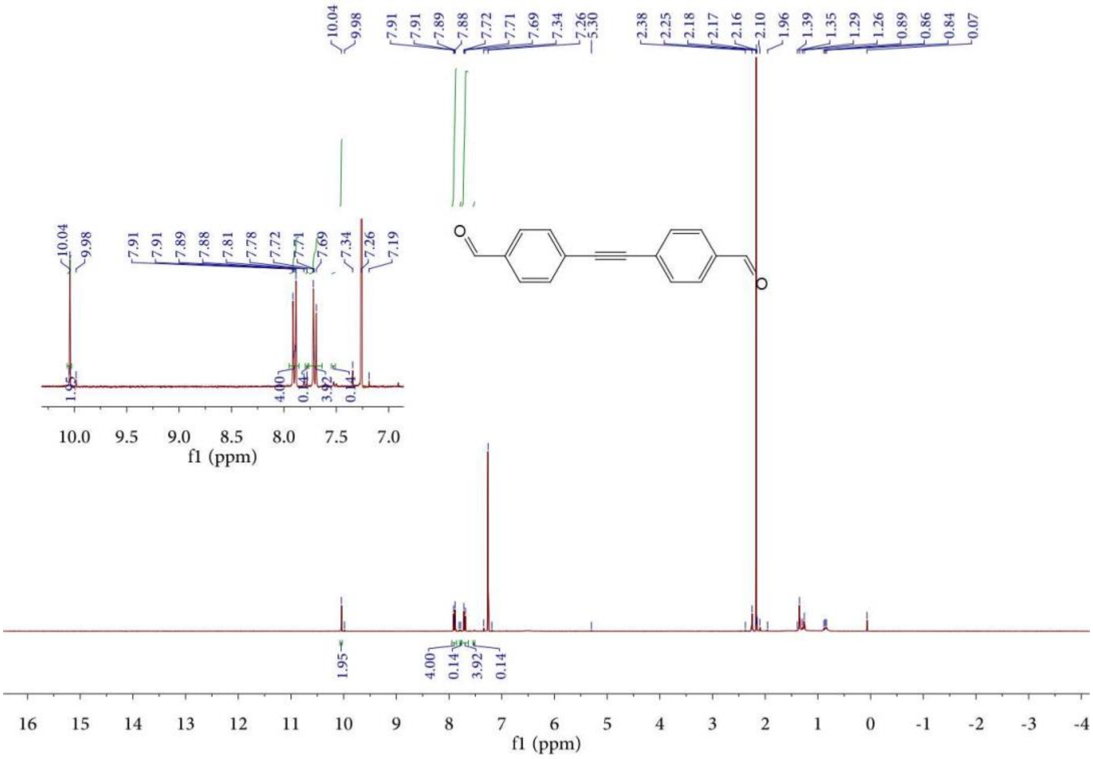
f1 (ppm)

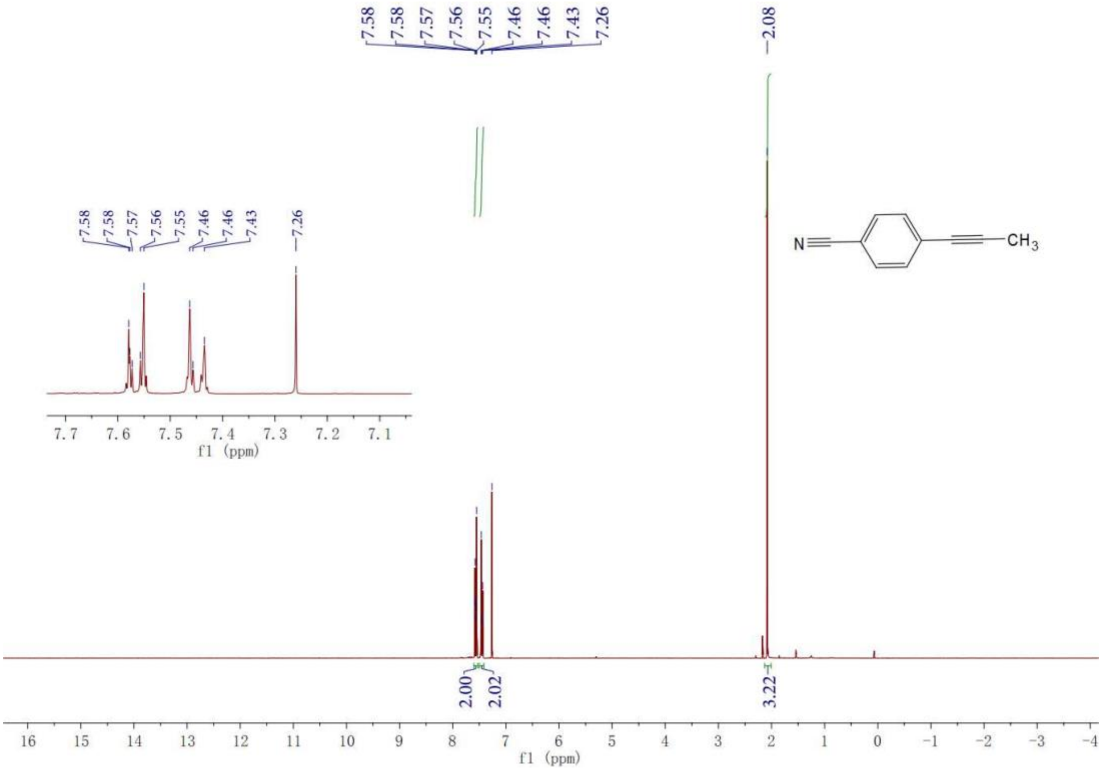

7.58 7.58 7.57 7.56 7.55 7.46 7.46 7.43 7.26
2.08
2.00
2.02
3.22
f1 (ppm)

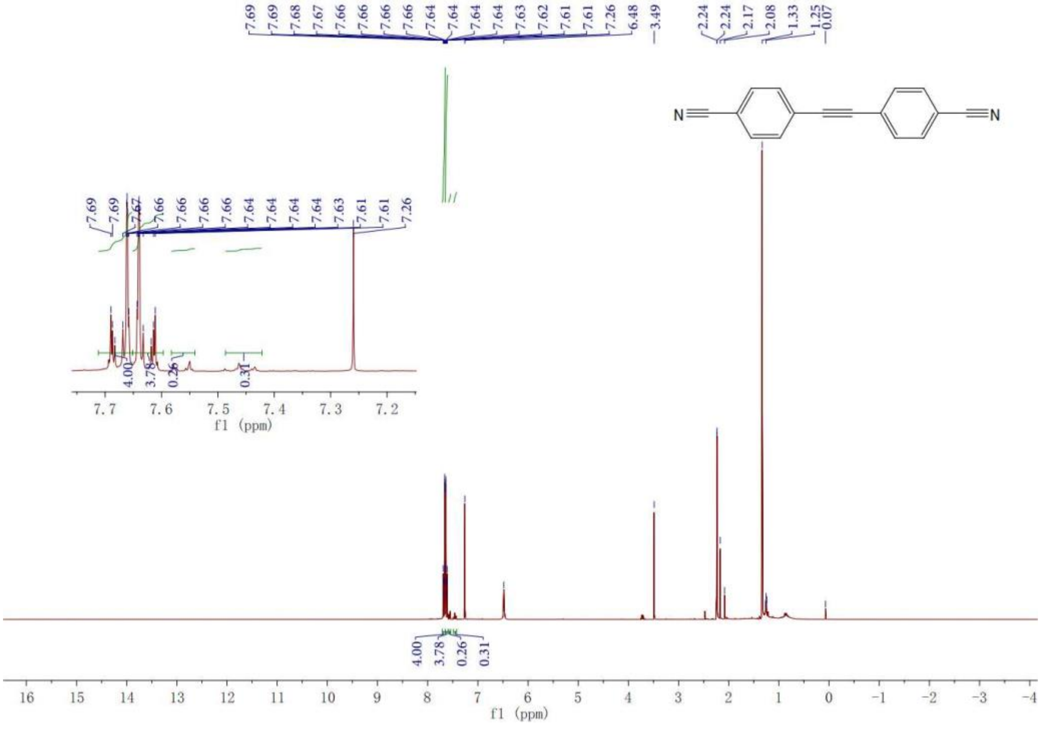

7.69 7.69 7.68 7.67 7.66 7.66 7.66 7.66 7.64 7.64 7.64 7.64 7.63 7.62 7.61 7.61 7.26 6.48
3.49
2.24 2.24 2.17 2.08 1.33 1.25 0.07
4.00
3.78
0.26
0.31
f1 (ppm)

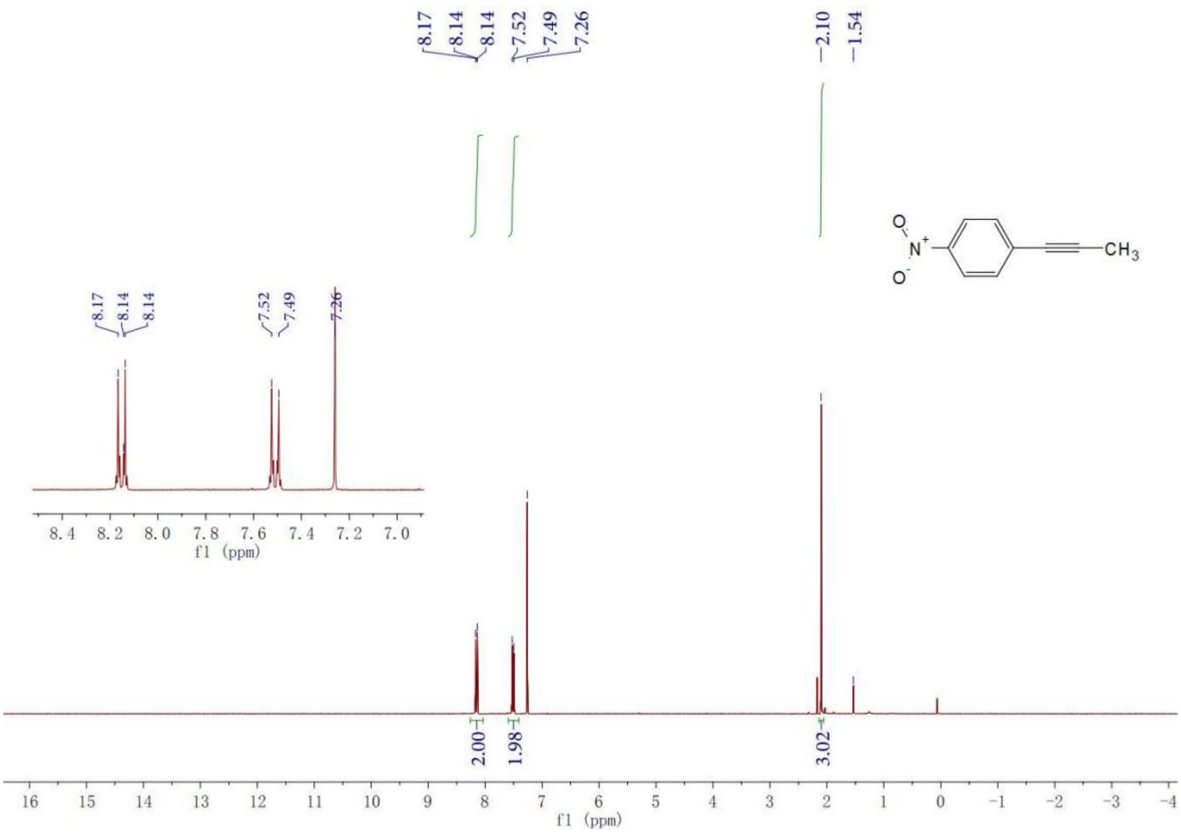
8.17
8.14
8.14
7.52
7.49
7.26
2.10
1.54
CH_3
2.00
1.98
3.02
f1 (ppm)

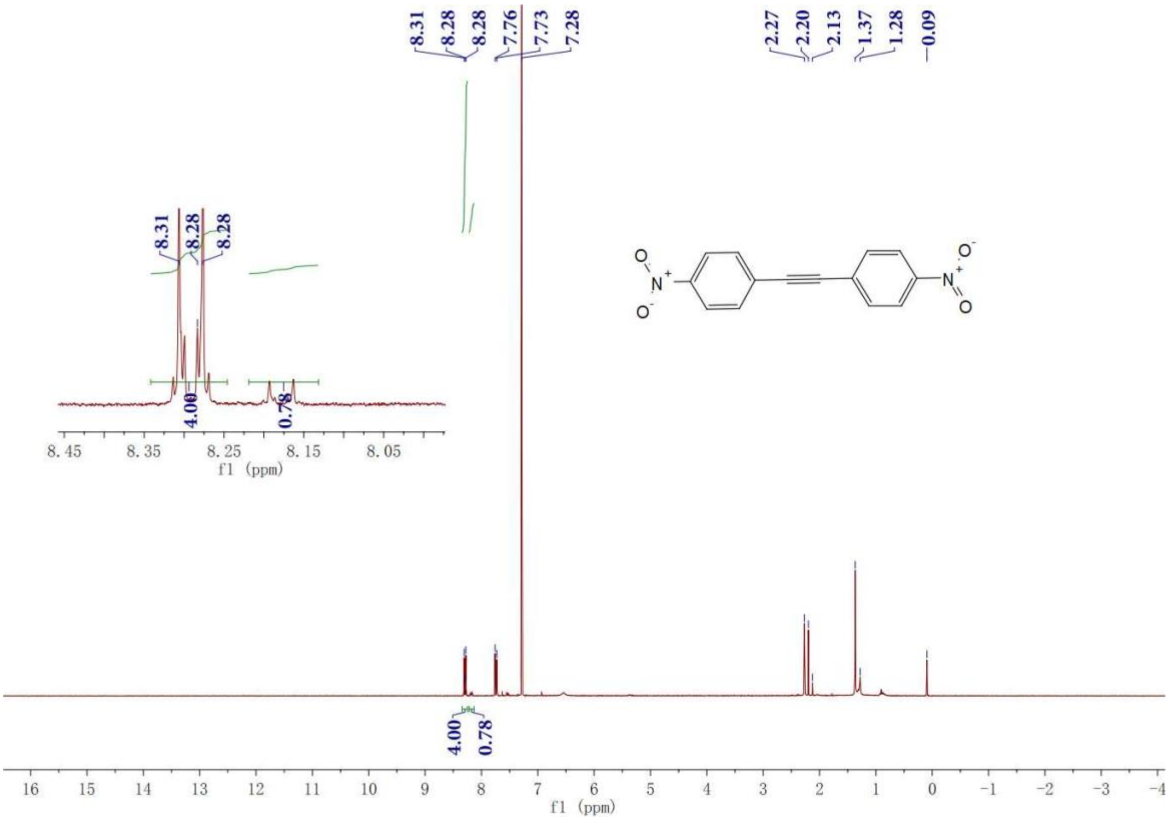
8.31
8.28
8.28
7.76
7.73
7.28
2.27
2.20
2.13
1.37
1.28
0.09
4.00
0.78
f1 (ppm)

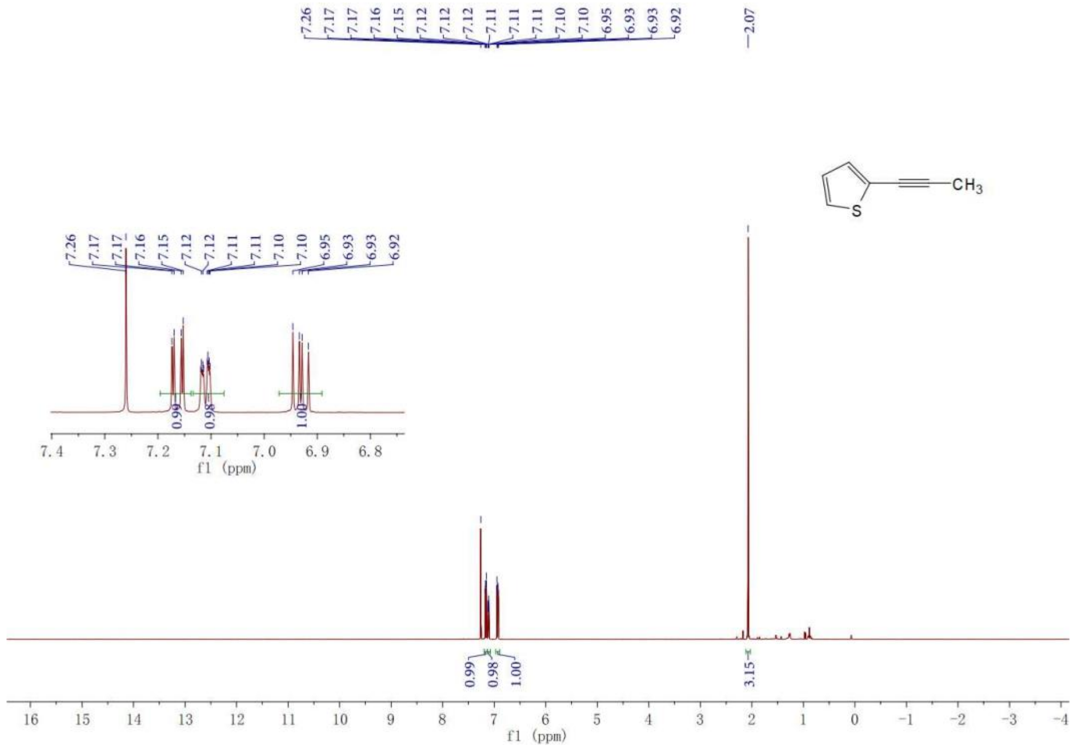

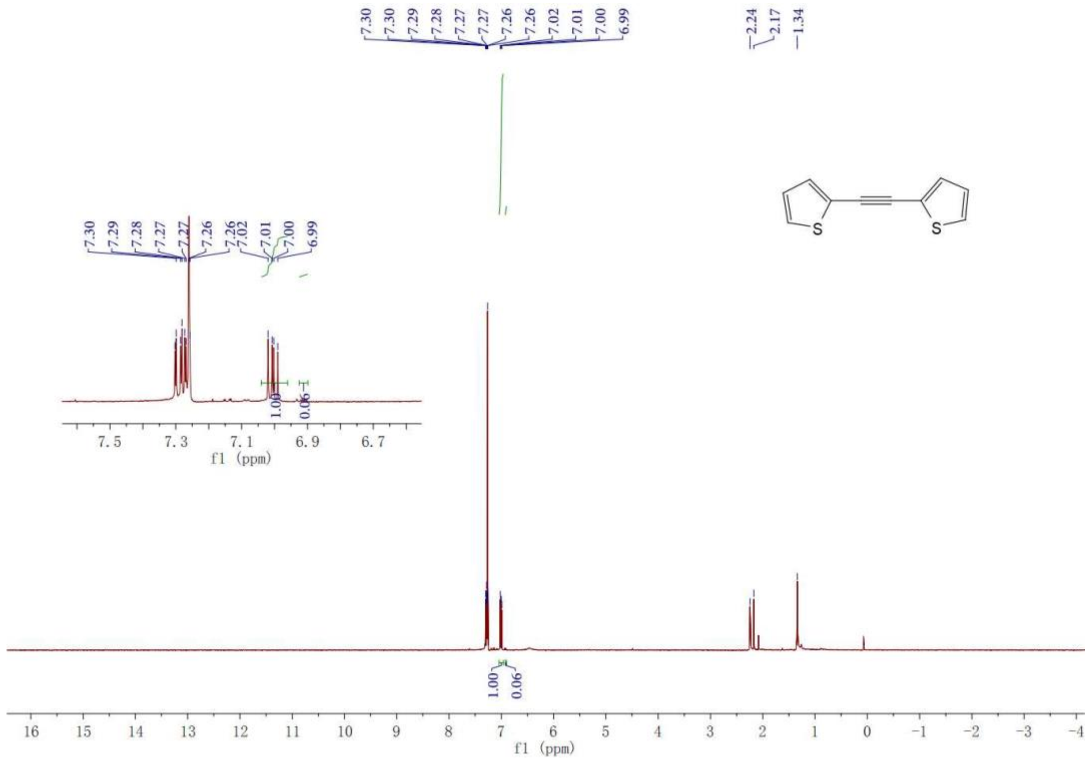

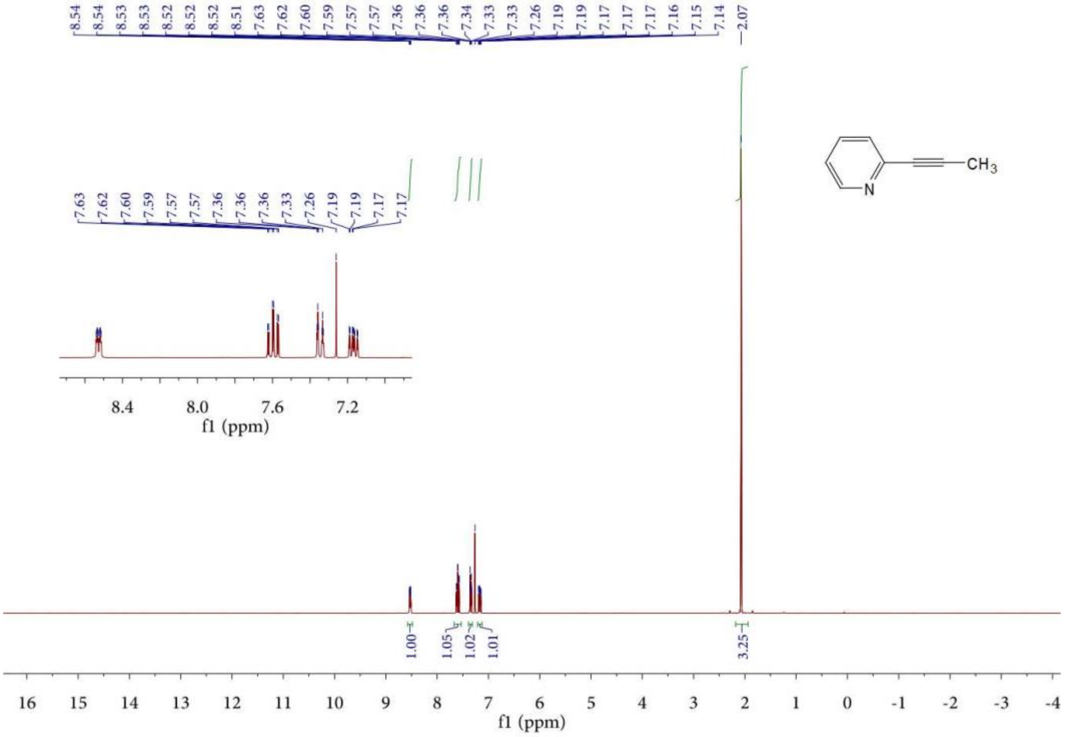
CH3
N
f1 (ppm)

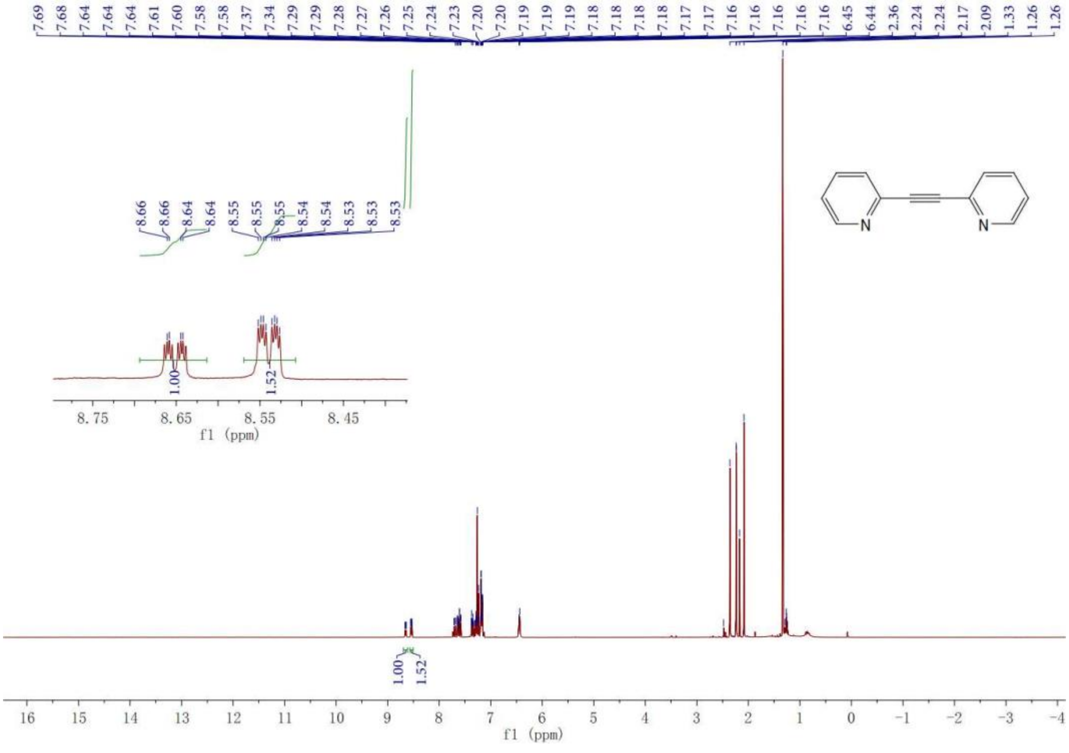
N
N
f1 (ppm)

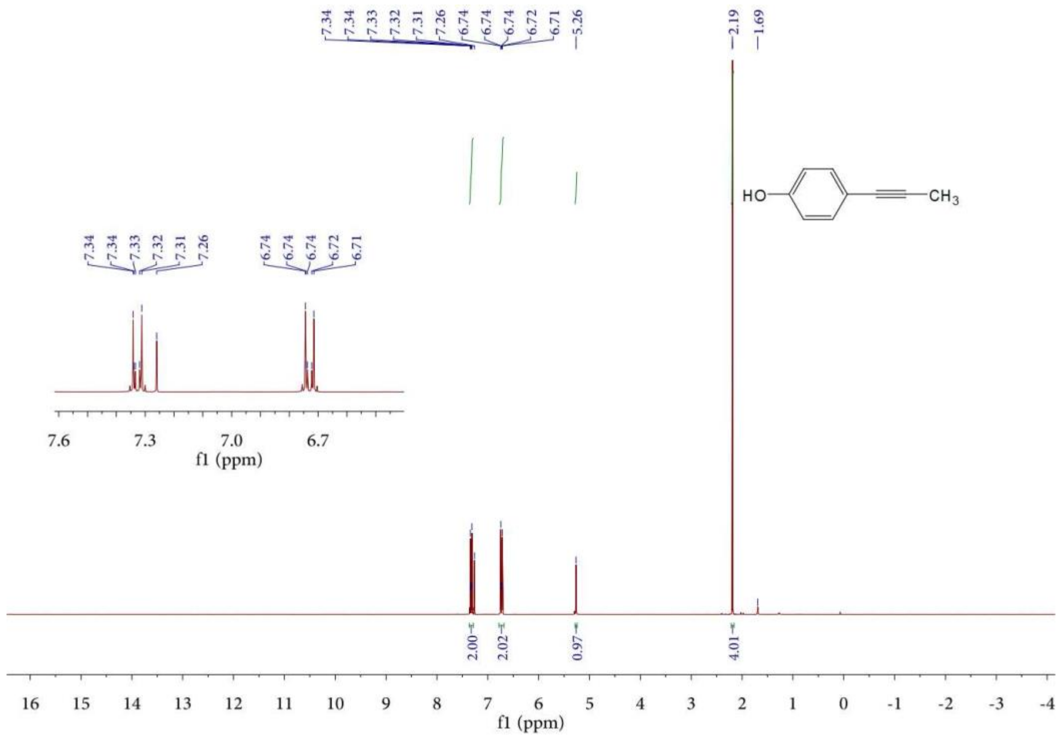

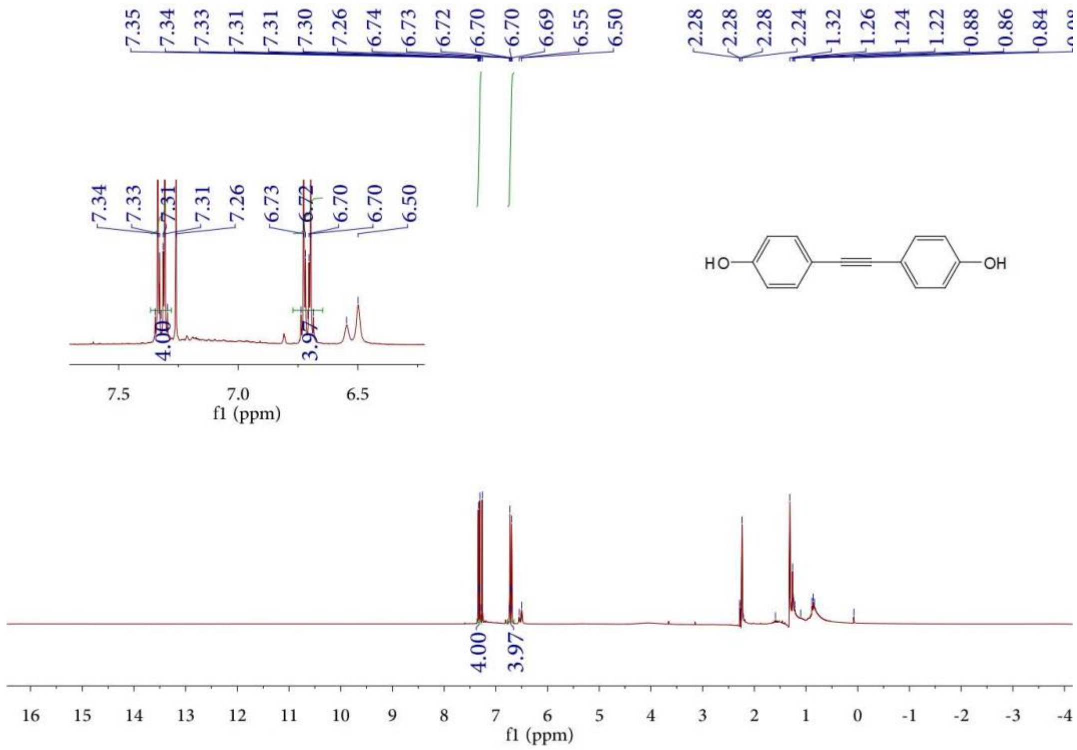

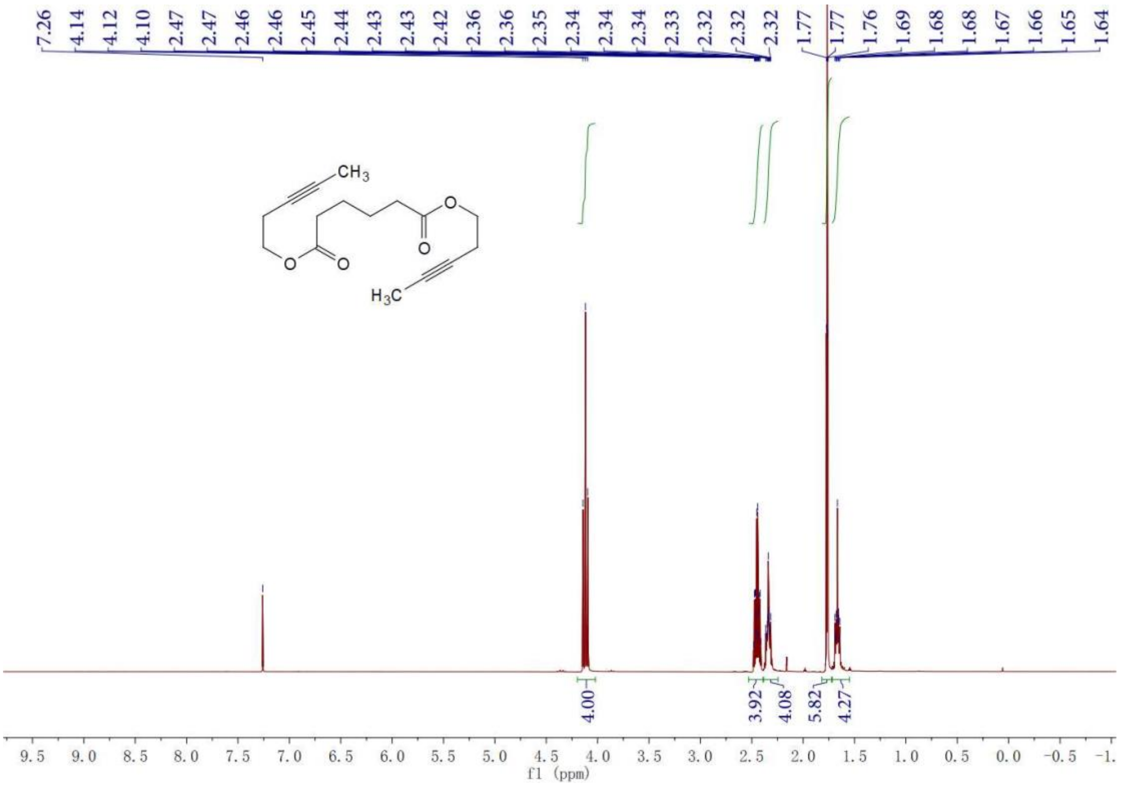
CH3
H3C
4.00
3.92
4.08
5.82
4.27
f1 (ppm)

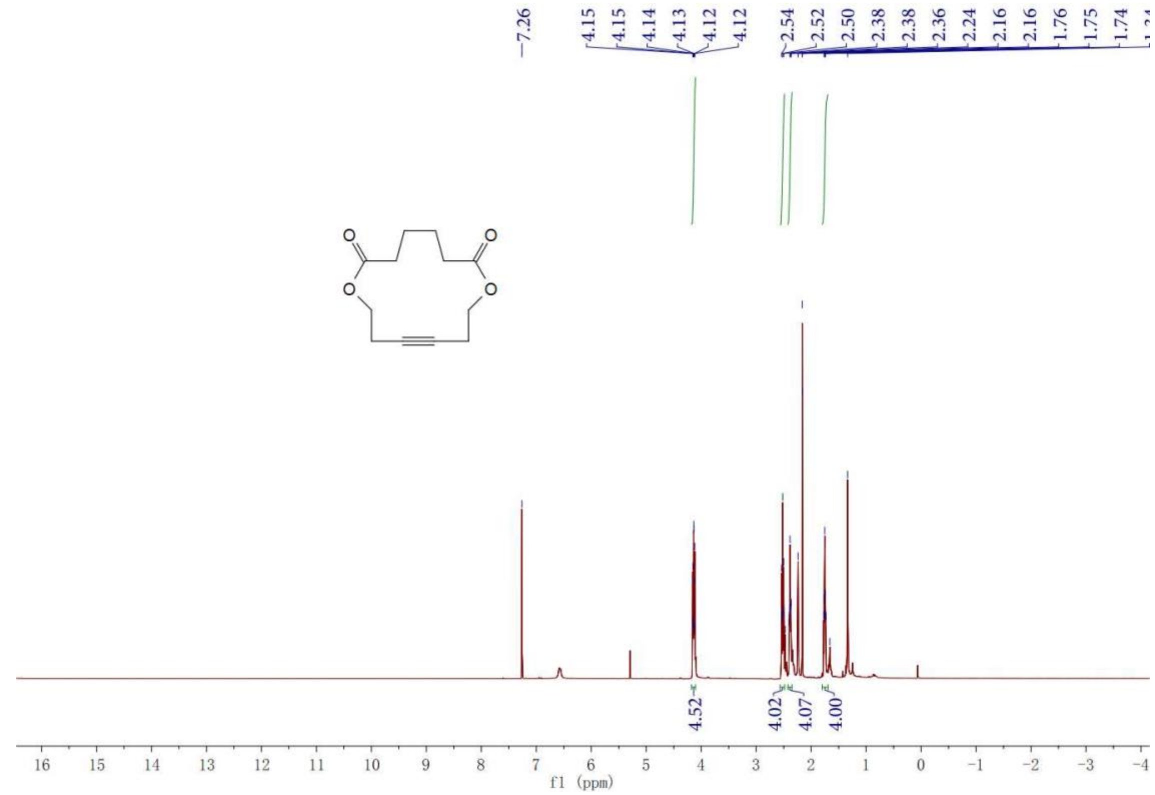
4.52
4.02
4.07
4.00
f1 (ppm)

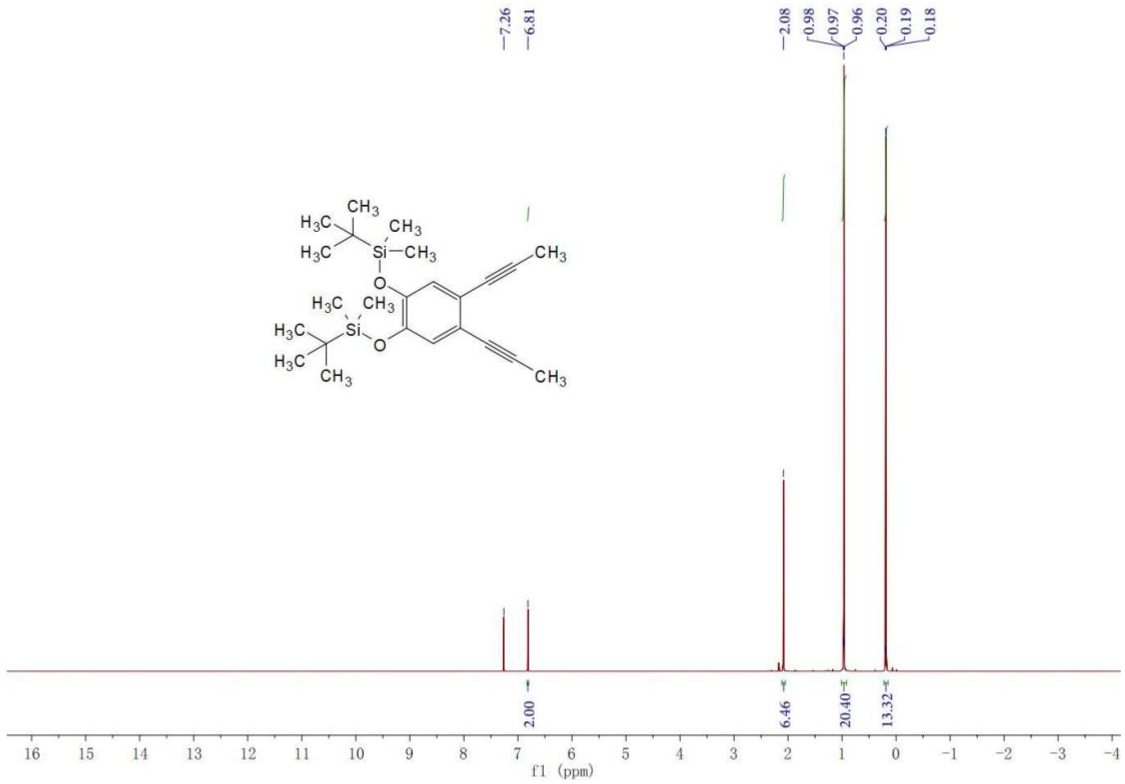
7.26
6.81
2.08
0.98
0.97
0.96
0.20
0.19
0.18
H_3C
CH_3
CH_3
H_3C
Si
CH_3
O
H_3C
CH_3
H_3C
Si
O
H_3C
CH_3
CH_3
CH_3
2.00
6.46
20.40
13.32
16 15 14 13 12 11 10 9 8 7 6 5 4 3 2 1 0 -1 -2 -3 -4
f1 (ppm)

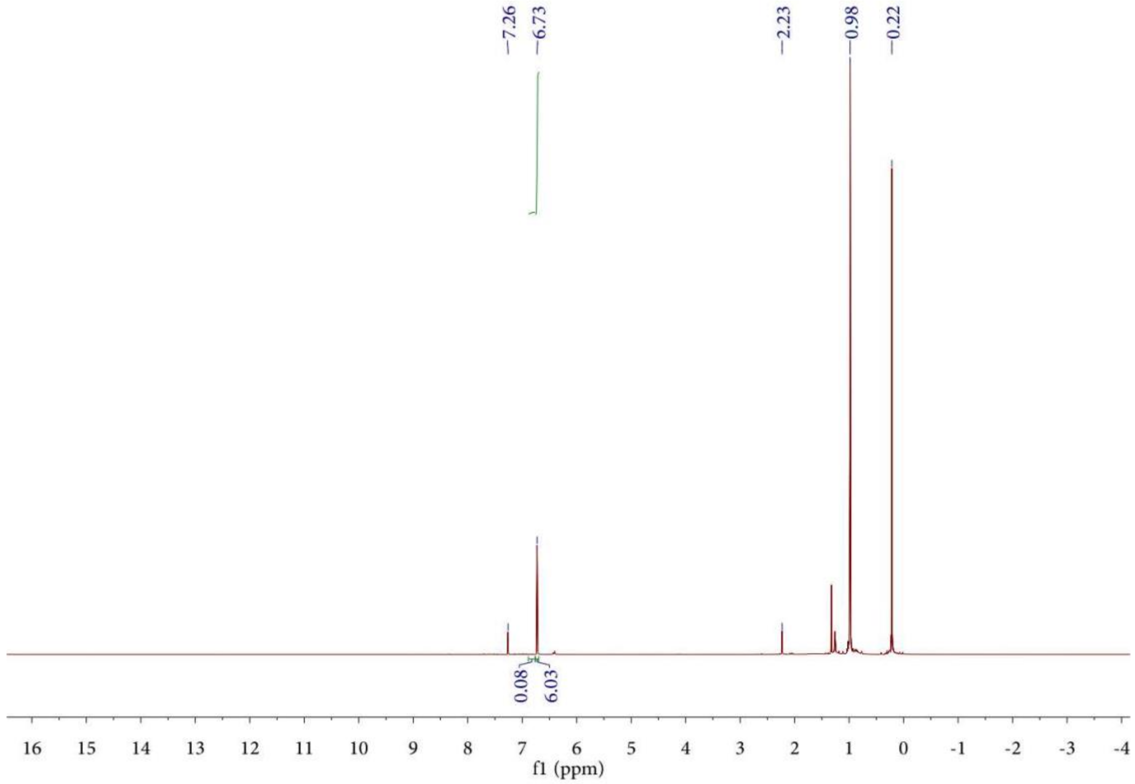
7.26
6.73
2.23
0.98
0.22
0.08
6.03
16 15 14 13 12 11 10 9 8 7 6 5 4 3 2 1 0 -1 -2 -3 -4
f1 (ppm)

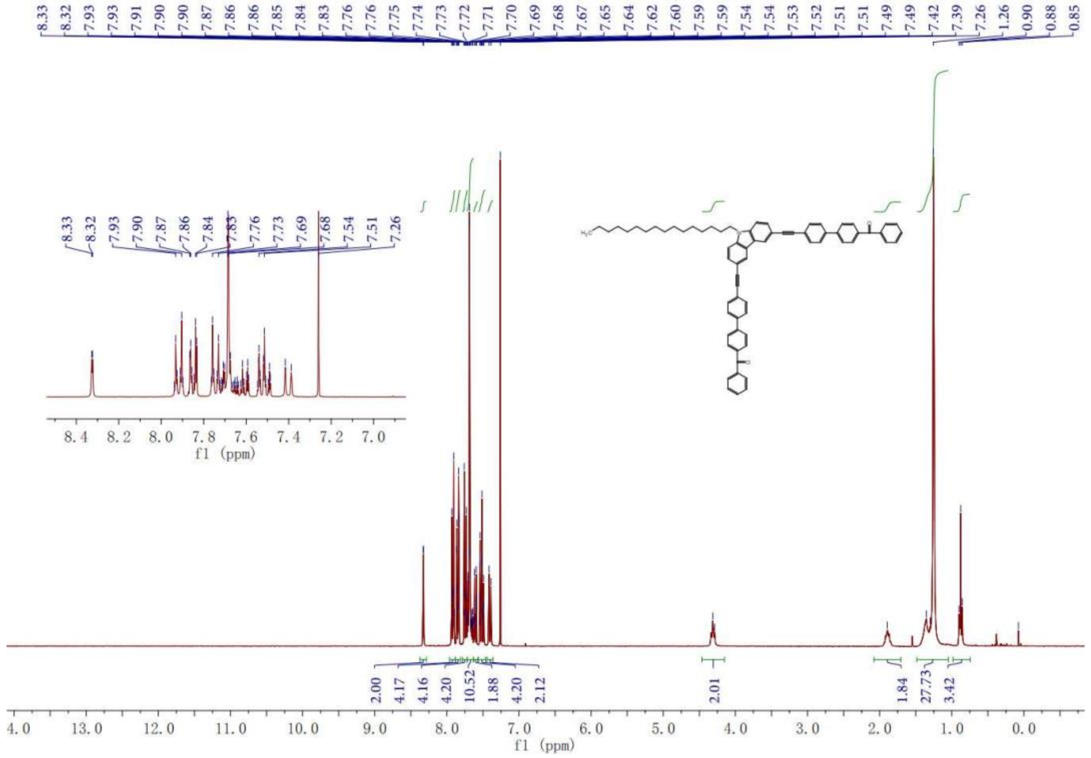

8.33 8.32 7.93 7.93 7.91 7.90 7.90 7.87 7.86 7.86 7.85 7.84 7.83 7.76 7.76 7.75 7.74 7.73 7.72 7.71 7.70 7.69 7.68 7.67 7.65 7.64 7.62 7.60 7.59 7.59 7.54 7.54 7.53 7.52 7.51 7.51 7.49 7.49 7.42 7.39 7.26 1.26 0.90 0.88 0.85
8.33 8.32 7.93 7.90 7.87 7.86 7.84 7.83 7.76 7.73 7.69 7.68 7.54 7.51 7.26
8.4 8.2 8.0 7.8 7.6 7.4 7.2 7.0
f1 (ppm)
2.00 4.17 4.16 4.20 10.52 1.88 4.20 2.12 2.01 1.84 27.73 3.42
4.0 13.0 12.0 11.0 10.0 9.0 8.0 7.0 6.0 5.0 4.0 3.0 2.0 1.0 0.0
f1 (ppm)

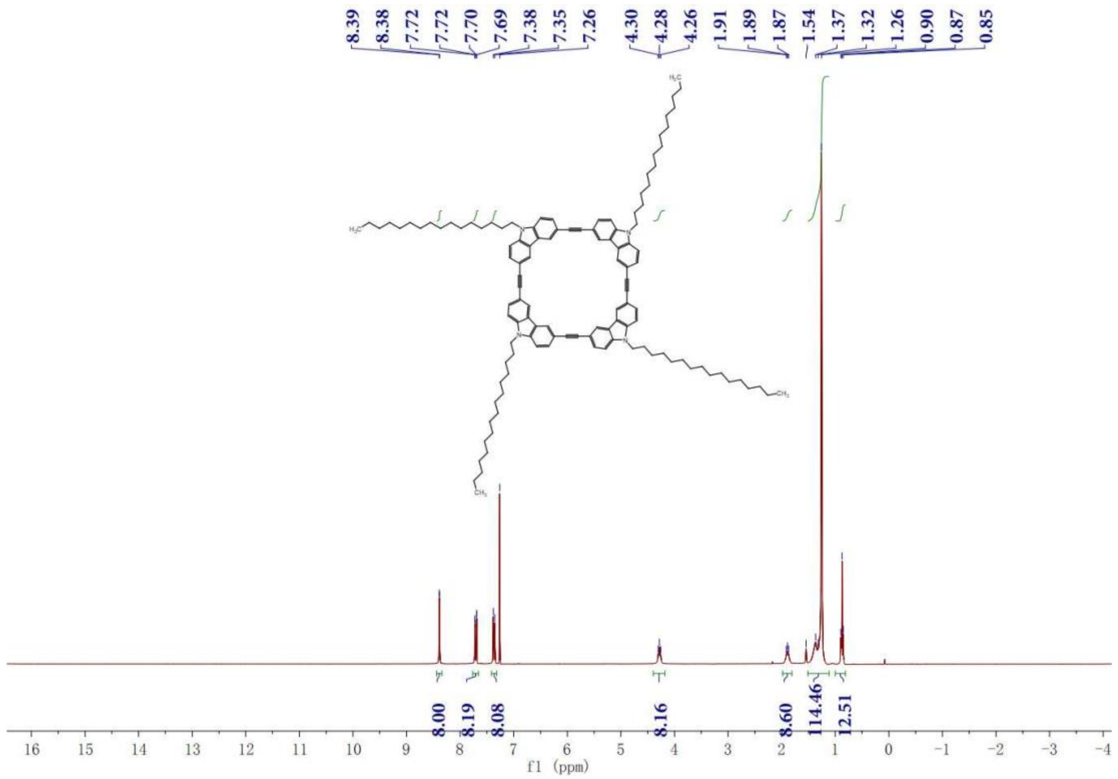

8.39 8.38 7.72 7.72 7.70 7.69 7.38 7.35 7.26 4.30 4.28 4.26 1.91 1.89 1.87 1.54 1.37 1.32 1.26 0.90 0.87 0.85
8.00 8.19 8.08 8.16 8.60 114.46 12.51
16 15 14 13 12 11 10 9 8 7 6 5 4 3 2 1 0 −1 −2 −3 −4
f1 (ppm)

10. X-ray Crystal Structure Analysis of Compound 1d (CCDC1974825)

Formula	$C_{22}H_{13}N_3O_3$
fw, g/mol	367.35
Temperature	100 K
cryst. syst.	monoclinic
space group	C 1 2/c 1
color	colorless
a (Å)	18.789(2)
b (Å)	12.9400(15)
c (Å)	22.147(3)
α (deg)	90
β (deg)	104.876(4)
γ (deg)	90
V ($Å^3$)	5204.1(11)
Z	8
h, k, l_{max}	25, 17, 29
N ref	6763
Tmin,Tmax	0.710, 0.746
Data completeness	0.998
*w*R2 (reflections)	0.2837(6763)
R(reflections)	0.0757(5572)
S	1.121

The X-ray crystal structure of **VIII-1d** was collected on a Bruker D8 Quest Eco three circle goniometer platform equipped with a Bruker Photon II detector. A graphite monochromator was employed for wavelength selection of the Mo Kα radiation (λ = 0.71073 Å). An Oxford Cryostream 800 was used to cool the crystal to 100 K during the data collection. The data were processed using APEX III software provided by Bruker. The structure was solved by intrinsic phasing in SHELXT[6] and refined by standard difference Fourier techniques with SHELXL[7] within the OLEX2[8] software package. All C-H hydrogen atoms were placed in calculated positions using the standard riding model. The O-H protons were located and placed from the Fourier difference map, and refined semi-freely using the DFIX command. Two of the three nitrile groups were found to be disordered over two positions above and below the plane of the parent phenyl ring. The occupancies of one disordered nitrile group were refined to 0.76(5)/0.24(5), while the occupancies of the two components of the other disordered nitrile group were fixed to 0.5/0.5. A highly disordered solvent molecule was present in the lattice and tentatively identified as ethyl acetate. The contribution of the disordered solvent to the diffraction pattern was removed using the OLEX2 implementation of SQUEEZE[9]. One of the phenolic O-H groups is missing a hydrogen bond acceptor, likely due to the removal of the disordered ethyl acetate molecule from the model.

11. Supplementary References

1. Zhang, W.; Lu, Y.; Moore, J. S. *Org. Synth*. **2007**, *84*, 163-176.
2. Heppekausen, J.; Stade, R.; Goddard, R.; Fürstner, A. *J. Am. Chem. Soc.* **2010**, *132*, 11045-11057.
3. Du, Y.; Yang, H.; Zhu, C.; Ortiz, M.; Okochi, K. D.; Shoemaker, R.; Jin, Y.; Zhang, W. *Chem. Eur. J.* **2016**, 22, 7959-7963.
4. Yasuda, M.; Yoshioka, S.; Yamasaki, S.; Somyo, T.; Chiba, K.; Baba, A. *Org. Lett.* **2006**, *8*, 761-764.
5. Yasuda, M.; Nakajima, H.; Takeda, R.; Yoshioka, S.; Yamasaki, S.; Chiba, K.; Baba, A. *Chem. Eur. J.* **2011**, *17*, 3856-3867.
6. Sheldrick, G. M. SHELXT – Integrated Space-Group and Crystal-Structure Determination. *Acta Crystallogr. Sect. Found. Adv.* **2015**, *71*, 3–8.
7. Sheldrick, G. M. Crystal Structure Refinement with SHELXL. *Acta Crystallogr. Sect. C Struct. Chem.* **2015**, *71*, 3–8.
8. Dolomanov, O. V.; Bourhis, L. J.; Gildea, R. J.; Howard, J. a. K.; Puschmann, H. OLEX2: A Complete Structure Solution, Refinement and Analysis Program. *J. Appl. Crystallogr.* **2009**, *42*, 339–341.
9. Spek, A. L. PLATON SQUEEZE: A Tool for the Calculation of the Disordered Solvent Contribution to the Calculated Structure Factors. *Acta Crystallogr. Sect. C Struct. Chem.* **2015**, *71*, 9–18.
